# Evaluation of Functional Properties of Some Lactic Acid Bacteria Strains for Probiotic Applications in Apiculture

**DOI:** 10.3390/microorganisms12061249

**Published:** 2024-06-20

**Authors:** Adriana Cristina Urcan, Adriana Dalila Criste, Otilia Bobiș, Mihaiela Cornea-Cipcigan, Alexandru-Ioan Giurgiu, Daniel Severus Dezmirean

**Affiliations:** 1 Department of Microbiology and Immunology, Faculty of Animal Science and Biotechnologies, University of Agricultural Sciences and Veterinary Medicine, 400372 Cluj-Napoca, Romania; adriana.urcan@usamvcluj.ro (A.C.U.); adriana.criste@usamvcluj.ro (A.D.C.); 2 Department of Apiculture and Sericulture, Faculty of Animal Science and Biotechnologies, University of Agricultural Sciences and Veterinary Medicine, 400372 Cluj-Napoca, Romania; obobis@usamvcluj.ro (O.B.); ddezmirean@usamvcluj.ro (D.S.D.); 3 Department of Horticulture and Landscaping, Faculty of Horticulture, University of Agricultural Sciences and Veterinary Medicine, 400372 Cluj-Napoca, Romania; mihaiela.cornea@usamvcluj.ro

**Keywords:** lactic acid bacteria, *Lactiplantibacillus plantarum*, *Lactobacillus acidophilus*, *Apilactobacillus kunkeei*, probiotics for bees, bee pathogens

## Abstract

This study evaluates the suitability of three lactic acid bacteria (LAB) strains—*Lactiplantibacillus plantarum*, *Lactobacillus acidophilus*, and *Apilactobacillus kunkeei*—for use as probiotics in apiculture. Given the decline in bee populations due to pathogens and environmental stressors, sustainable alternatives to conventional treatments are necessary. This study aimed to assess the potential of these LAB strains in a probiotic formulation for bees through various in vitro tests, including co-culture interactions, biofilm formation, auto-aggregation, antioxidant activity, antimicrobial activity, antibiotic susceptibility, and resistance to high osmotic concentrations. This study aimed to assess both the individual effects of the strains and their combined effects, referred to as the LAB mix. Results indicated no mutual antagonistic activity among the LAB strains, demonstrating their compatibility with multi-strain probiotic formulations. The LAB strains showed significant survival rates under high osmotic stress and simulated gastrointestinal conditions. The LAB mix displayed enhanced biofilm formation, antioxidant activity, and antimicrobial efficacy against different bacterial strains. These findings suggest that a probiotic formulation containing these LAB strains could be used for a probiotic formulation, offering a promising approach to mitigating the negative effects of pathogens. Future research should focus on in vivo studies to validate the efficacy of these probiotic bacteria in improving bee health.

## 1. Introduction

Bees, essential pollinators in agricultural ecosystems, play a critical role in maintaining biodiversity and ensuring crop production. However, lately, bee populations worldwide are facing significant declines, a phenomenon often attributed to a complex interplay of various diseases and environmental stressors [1,2,3]. Pathogenic diseases, including viral, bacterial, fungal, and parasitic infections, significantly contribute to this decline [4,5,6,7]. Viruses like the deformed wing virus, often transmitted by parasitic mites such as *Varroa destructor* [8], severely weaken bee colonies. Bacterial infections, notably American Fulbrood caused by *Paenibacillus larvae* [9] and European Foulbrood caused by *Melissococcus plutonius*, and other associated bacterial flora consisting of *Paenibacillus alvei*, *Enterococcus faecalis*, and *Bacterium eurydice*, lead to substantial brood mortality [10,11]. Fungal pathogens, especially *Nosema ceranae*, cause nosemosis, impacting bees’ digestive tracts [12,13], while chalkbrood caused by *Ascosphaera apis* affects larvae [14]. Additionally, *Varroa destructor* mites weaken bees by feeding on their hemolymph and spreading various viruses, leading to conditions like varroosis [15]. Moreover, environmental stressors further exacerbate these issues [16]; pesticide exposure, particularly from neonicotinoids, impairs bees’ behavior and immune response [17,18]. Habitat loss and climate change reduce forage availability and disrupt natural foraging patterns. Addressing bee population decline requires understanding the interactions between these diseases and stressors [19,20]. Comprehensive research and multidisciplinary strategies are essential for protecting bee health, maintaining biodiversity, and ensuring the sustainability of agricultural systems worldwide. Conventional treatments for bee diseases include antibiotics, antifungals, and acaricides. However, these treatments, while effective, can lead to resistance and residues in bee products, prompting the need for sustainable alternatives [21].

In recent years, researchers have focused on the bee microbiome and the use of lactic acid bacteria (LAB) as a sustainable approach to prevent and treat honeybee diseases. The honeybee gut microbiota is composed of a large variety of bacteria, including numerous LAB within the genera *Lactobacillus* as well as bacteria from the genus *Bifidobacterium*, which serves as a defense mechanism against pathogen infections [22,23]. Probiotics have been shown to improve gut microbiota balance, enhance bees’ resistance to nosemosis, and reduce mortality rates [24,25]. Additionally, probiotics have shown promise in mitigating the effects of American Foulbrood and European Foulbrood, both caused by pathogenic bacteria [26,27,28], improving brood survival and colony strength [29,30]. *Apilactobacillus* has been found to enhance the immune system of bees by significantly increasing the synthesis of vitellogenin in their fat bodies. This lipoprotein is essential for immune responses, longevity, and stress resistance. The defined gut microbiota further boosts vitellogenin transcription, underscoring the microbiota’s crucial role in bee health [31]. Recent studies [32,33] revealed that *Lactobacillus kunkeei* enhances honey bee (*Apis mellifera*) survival against *Serratia marcescens* by inhibiting its proliferation in the gut and modulating immune responses. *L. kunkeei* reduces the expression of antimicrobial peptides like apidaecin, abaecin, and hymenoptaecin, preventing excessive immune activation, thereby maintaining gut homeostasis and protecting against opportunistic pathogens. Other authors [34] reported that isolates of *L. kunkeei* from the honey bee gut microbiota can inhibit the growth of *Paenibacillus larvae* and *Nosema ceranae* both in vitro and in vivo. The administration of these isolates increased larval viability and reduced mortality associated with *P. larvae* infection. Furthermore, *Lactobacillus* strains showed antimicrobial properties against *P. larvae and* demonstrated potential as probiotics due to their high adhesion, safety to larvae, and inhibition of *P. larvae* [26].

However, the efficacy of probiotic treatments can vary depending on the strain used and the specific health condition of the bee colony [35], but overall, probiotics represent a promising avenue for improving bee health and resilience against diseases, contributing to the sustainability of bee populations and agriculture [36]. Studies show that bees’ microbiota is directly related to food sources [37], geographic region, age, the state of health of the bees, and the use of antibiotics [38,39,40].

LAB exhibits properties like the ability to synthesize antimicrobial peptides that can cause cell lysis or enzyme inhibition, produce organic acids, metabolize different sugars, and increase the number of phenolic compounds [41,42]. These features explain the effectiveness of LAB in colonizing the sugar-rich digestive system of bees and suggest a potential for inhibiting the growth of pathogens [43]. The significant function of gut microorganisms in maintaining digestive health, honeybee well-being, and immunity could offer a potential approach to addressing different bees’ pathogens, like *N. cerane*. For these reasons, treatments with probiotics and prebiotics have gained attention as potential methods for mitigating the negative effects of bee pathogens [44,45,46]. Previous research has demonstrated the ability of certain probiotic strains to improve the intestinal health of bees and reduce the incidence of infections [24,47].

The purpose of this study was to evaluate the potential of a mixture of three bacterial strains—*Lactiplantibacillus plantarum*, *Lactobacillus acidophilus*, and *Apilactobacillus kunkeei* (LAB mix)—for use in a probiotic product for honeybees. The objectives included testing the biocompatibility of these strains when used together, assessing their resistance to high osmotic concentrations, and observing their combined effects. This study also involved analyzing co-culture interactions, biofilm formation, auto-aggregation, antioxidant activity, antimicrobial activity, antibiotic susceptibility, and mutual antagonistic activity using the agar slab method. These comprehensive evaluations aimed to determine the suitability and efficacy of these strains as probiotics, contributing to the development of a probiotic formulation to enhance bee health and resilience.

## 2. Materials and Methods

### 2.1. Bacteria Strains

*Lactobacillus plantarum* ATCC 8014, *Lactobacillus acidophilus* ATTC 700396, and *Apilactobacillus kunkeei* ATCC 700308 were employed in this study. Pathogenic bacteria for bees were used, such as *Enterococcus faecalis* ATCC 29212, *Paenibacillus larvae* ATCC 9545, *Melissococcus plutonius* ATCC 35311, and *Paenibacillus alvei* ATCC 6344, but also other pathogenic bacteria that are not among the common pathogens of bees, like *Staphylococcus aureus* ATCC 25923, *Bacillus cereus* ATCC 11778, *Escherichia coli* ATCC 25922, *Pseudomonas aeruginosa* ATCC 27853, *Salmonella enteritidis* ATCC 13076, and yeast *Candida albicans* ATCC 10231. Before being used, each strain was grown at 37 °C in the proper conditions and stored at −80 °C in glycerol.

### 2.2. Co-Culture of L. plantarum, A. kunkeei, and L. acidophilus

This test was performed following the protocol described by [48] with slight modifications. Bacterial cells from 24 h LAB cultures at 37 °C were harvested by centrifuging at 8000 rpm for 10 min at 4 °C, washed twice, and resuspended in sterile saline to an optical density of ~0.5 McFarland at 600 nm. Individual LAB suspensions of *L. plantarum*, *A. kunkeei*, and *L. acidophilus* were inoculated (1% *v*/*v*) into MRS broth (Sigma Aldrich, Darmstadt, Germany) and incubated at 37 °C. For the co-culture (LAB mix), equal volumes (1% *v*/*v*) of each bacterial suspension were combined and vortexed to ensure thorough mixing. Microbial counts were performed on the LAB mix and single cultures at time 0 and after 4, 6, and 24 h of incubation.

### 2.3. Determination of Mutual Antagonistic Activity Using Agar Slab Method

LAB cultures with a density of 1.5 × 10^8^ CFU/mL (equivalent to 0.5 McFarland Standard) were spread onto Petri dishes containing MRS agar (Sigma Aldrich, Darmstadt, Germany) using a sterile cell spreader and incubated at 37 °C for 24 h. Three 10 mm diameter discs were cut from the solid medium and put on MRS agar plates inoculated with a different LAB strain. After that, the plates were incubated for a further 24 h at 37 °C. Following incubation, the test strain’s growth inhibition zone diameter was determined [49]. The following standards [50] were applied in order to compare the antagonistic activity of LAB strains: growth inhibition diameters larger than 11 mm were indicative of strong inhibition (+++), 6–10 mm indicated moderate inhibition (++), 1–5.9 mm indicated weak inhibition (+), and 0 mm indicated no inhibition (−).

### 2.4. Osmotic Stress Resistance Test—Viability of LAB in Syrup

To select the highest concentration of the syrup in which the LAB survives, inoculation of syrups at various concentrations ranging from 45% to 80% was carried out, followed by cultivation in both liquid and solid MRS medium. The two syrups used were freshly prepared sugar syrup (sucrose from Sigma Aldrich, Darmstadt, Germany) and a commercially available glucose + fructose syrup containing 53.6% glucose and 42.9% fructose, according to the manufacturer’s declaration. The positive control was represented by the natural growth curve of bacteria in the MRS medium, while the negative control was represented by the MRS medium. To observe the growth curves of LAB, the samples grown in liquid MRS were incubated at 35 °C for 48 h, and their absorbance was monitored spectrophotometrically at 600 nm every 30 min. Furthermore, the survival of LAB in the syrups was monitored over a period of 7 days to evaluate their longevity; during this period, the lactobacilli were put in contact with the sugar solution, or glucose + fructose syrup, and samples were taken every 24 h. The samples were diluted, grown on solid MRS, and incubated under the same conditions as for the liquid medium. After 48 h, the total number of colonies was counted and expressed in CFU/mL.

### 2.5. Viability of LAB in Simulated In Vitro Gastrointestinal Digestive Conditions

Given the absence of an in vitro gastrointestinal model for honeybees, this test adapted methods from existing studies [49,51,52], adjusting the composition of simulated gastric and intestinal juices to match honeybee digestive conditions. The process involved the cultivation of LAB in MRS broth for 24 h, followed by centrifugation at 2300× *g* for 15 min. The resultant pellet was repeatedly washed with a sterile 0.85% NaCl solution to remove all remnants of the culture medium. Subsequently, these washed cultures were resuspended in sugar syrups and standardized to achieve an optical density at 600 nm of 1.0 ± 0.05. The standardized LAB mixture was then introduced into a freshly prepared simulated gastric juice containing 0.85% NaCl, 100 U/mL α-amylase, and 0.15% pepsin (pH = 3.0). All reagents were of analytical grade and were purchased from Sigma Aldrich, Darmstadt, Germany. This mixture was incubated at 35 °C for two hours with gentle agitation at 80 rpm. Following this, the LAB suspension was combined with freshly prepared simulated intestinal juice comprising 1% bile salts, 0.1% pancreatin, and 0.85% NaCl, with the pH adjusted to 7.5. This new mixture was incubated for an additional two hours under the same conditions. After each incubation period, 1 mL of each sample was put into sterile 0.85% NaCl, mixed, diluted, and plated on agar plates with MRS using the Koch plate method. The plates were then incubated for 48 h, after which the colonies were counted, and the results were expressed in CFU/mL. This procedure was repeated at specific time points, initially at 0 h, after two hours in simulated gastric juice, and after two more hours in simulated intestinal juice, to assess the survivability of LAB through the simulated honeybee digestive process.

### 2.6. Biofilm Formation

Biofilm formation for LAB was evaluated under static conditions, following a modified method by O’Toole [53]. After overnight incubation in MRS broth, bacterial cells were obtained by centrifugation for 10 min at 4 °C at 8000 rpm, rinsed twice with PBS, and then resuspended in MRS broth. A triplicate of 200 µL of each suspension was added to a 96-well microtiter plate. Wells containing uninoculated MRS broth were used as negative controls. After incubating at 37 °C for 24 h, the plates were rinsed three times using sterile saline to clear away any unattached cells. The cells that were attached were fixed using 200 µL of 99% methanol, dried, stained with 2% Crystal Violet, and washed again. Cells were resuspended in 160 µL of 30% glacial acetic acid after air drying. OD was measured at 580 nm using a BioTek Synergy 2 multichannel spectrophotometer (BioTek Instruments, Winooski, VT, USA). The results are presented as means ± standard deviations.

### 2.7. Auto-Aggregation Assay

The auto-aggregation assay was conducted in compliance with the previously defined method by Collado et al. [54]. Standardized optical density (OD) was set at 0.5 ± 0.05. Following an overnight incubation period at 37 °C, OD measurements were made on these bacterial suspensions at 1, 2, 5, and 24 h. Using Formula (1), the auto-aggregation percentage [48] was calculated.

(1) $$\mathrm{Auto-aggregation}\;(\%)=(1-\frac{{OD}_{\mathrm{final}}}{{OD}_{0}})\times \;100$$

where OD_0_ is the absorbance at the start of the experiment, and OD_final_ is the absorbance found after 1, 2, 6, and 24 h.

### 2.8. Quantity of Lactic Acid

Potentiometry was employed to calculate titrable acidity in line with [55], with slight changes. An automated titrator (TitroLine 5000 with electrode A7780, Roth, Germany), 0.1 N NaOH standard solution, and 10 mL of each sample were used. The results were displayed as equivalents of NaOH/kg^−1^. The percentage of lactic acid was calculated [56] by utilizing Formula (2):

(2) $$\mathrm{Lactic}\;\mathrm{acid}\;\mathrm{content}=(\frac{V_{NaOH}\times C_{NaOH}\times 0.09}{m})\times 100$$

where V_NaOH_—the volume of NaOH used for titration; C_NaOH_—concentration of NaOH; V—volume of the sample; and 0.09—equivalent weight of lactic acid.

### 2.9. Antioxidant Activity

#### 2.9.1. DPPH Method

The scavenging activity of LAB samples against the 2,2-diphenyl-1-picrylhydrazyl radical (DPPH) was checked by means of a spectrophotometric method [57]. Briefly, 40 µL of LAB solution was added over 200 µL of DPPH solution (0.02 mg/mL). The absorbance of the samples was recorded at 517 nm after 15 min. DPPH radical scavenging (%) was determined based on the decrease in absorbance at 540 nm and calculated according to Formula (3):

(3) $$\mathrm{DPPH}\;\mathrm{radical}\;\mathrm{scavenging}\;\mathrm{activity}\;(\%)=(1-\frac{A_{s}}{A_{0}})\;\times \;100$$

where A_0_ is the blank’s absorbance at 517 nm, and A_s_ is the sample’s absorbance at the same wavelength.

#### 2.9.2. ABTS Method

The ABTS method test was conducted using the methodology described by Margaoan et al. [58]. The test reduces the radical ABTS^•+^ (2,2′-azino-bis (3-ethylbenzothiazoline-6-sulfonic acid)) to a colorless product by scavenging it. Prior to measurement, the ABTS^•+^ had been diluted with ethylic alcohol to attain an absorbance of 0.700 ± 0.025 at 734 nm. For the assay, 30 µL of every sample was combined with 170 µL of the dilutedABTS^•+^ solution. The ABTS radical scavenging (%) was monitored by measuring absorption at 734 nm, calculated according to Formula (4):

(4) $$\mathrm{ABTS}\;\mathrm{radical}\;\mathrm{scavenging}\;\mathrm{activity}\;(\%)=(1-\frac{A_{s}}{A_{0}})\;\times \;100$$

where A_0_ is the blank’s absorbance at 517 nm and A_s_ is the sample’s absorbance at the same wavelength.

#### 2.9.3. FRAP Method

The method described by Cornea-Cipcigan et al. [59] was used to determine ferric reducing/antioxidant power with slight adjustments. A total of 300 µL of FRAP solution was mixed with 10 µL of each sample and 10 µL of ultrapure water. After that, samples underwent incubation for five minutes at 37 °C. The antioxidant capacity was determined by comparing the absorbance to a standard calibration curve (R^2^ = 0.997) constructed with known quantities of Fe^II^ solutions (0.1–1 mmol/L of FeSO_4_·7H_2_O). Absorbance was measured at 593 nm, and the results were reported as mmol of Fe^II^ per mL of sample.

### 2.10. Antimicrobial Activity

In the antimicrobial activity, supernatants with the metabolites of the lactic cultures were used; therefore, the 24 h cultures of bacteria grown in MRS were centrifuged (10,733× *g* for 15 min) to be separated from the cells. Further, the supernatants were used at the physiological pH (ranging from 3.81 to 4.51, depending on the strain). The supernatants were filtered through sterile syringe filters with a pore diameter of 0.22 μM.

#### 2.10.1. Disk Diffusion Method

Bacterial suspensions were adjusted to 0.5 McFarland, and 0.5 mL of each solution was added to Petri dishes with MH agar for bacteria and SDA agar for *Candida* spp. After removing excess liquid, the agar was dried at 37 °C for 15–20 min. Wells were created, and 30 µL of each sample was added. Positive controls included amoxicillin (30 μg/mL) for Gram-positive bacteria, norfloxacin (10 μg/mL) for Gram-negative bacteria, and miconazole (10 μg/mL) for yeast. The sizes of the inhibitory zones were measured in millimeters after the plates were incubated for 24 h at 37 °C for bacterial cultures and 48 h at 28 °C for fungal cultures. All tests were performed in triplicate.

#### 2.10.2. Microplate Assay

The following LAB supernatant concentrations were employed in the assays, which were conducted in 96-well plates: 50%, 25%, 12.5%, and 6.25%. Each well contained a total liquid volume of 200 µL and was inoculated with a final pathogen density of 1.5 × 108 CFU/mL. A BioTek Synergy 2 multichannel spectrophotometer (BioTek Instruments, Winooski, VT, USA) was used to measure the initial absorbance (0 h) at 600 nm. The plates were incubated for 24 h at 37 °C, after which the absorbance was measured again. The negative controls for the experiment were pathogens grown in a convenient medium. All tests were conducted in triplicate. The equation below (5) was used to measure the inhibition of bacterial growth (%), which is as follows:

(5) $$\mathrm{growth}\;(\%)=100-\frac{A_{24}}{A_{c}}\times 100$$

where A_24_ was the mean of three repetitions of the absorbance measurements after 24 h and A_c_ was the mean of three repetitions of the absorbance values of the negative control after 24 h.

### 2.11. Antibiotics Susceptibility Assay

LAB’s antibiotic susceptibility was assessed by the application of the disc diffusion technique. Using a sterile cell spreader, LAB cultures that had been cultivated overnight and corrected to a 0.5 McFarland density were placed on Petri plates filled with MRS agar. Antibiotic-impregnated paper discs were then placed in triplicate on each plate, which were subsequently incubated at 37 °C for 24 h. The antibiotics tested included: ampicillin (10 µg), chloramphenicol (50 µg), erythromycin (30 µg), tylosin (30 µg), tetracycline (30 µg), oxytetracycline (30 µg), streptomycin (25 µg), kanamycin (30 µg), gentamicin (30 µg), sulfonamides (300 µg), lincomycin (2 and 15 µg), and vancomycin (30 µg). Following incubation, the microbial growth inhibition zone’s diameter was measured and noted in millimeters. The antibiotic susceptibility profile of LAB was interpreted following established guidelines [60], and the susceptibility was classified as susceptible (S), moderately susceptible (MS), or resistant (R).

### 2.12. Statistical Analysis

The data obtained were analyzed using the analysis of variance (ANOVA). When the null hypothesis was rejected, post hoc tests were performed to determine significant differences between the means. Tukey’s HSD test was performed to compare the means and identify statistically significant differences for normally distributed data (*p* < 0.05). To assess the survival rates over the study period, Kaplan–Meier survival curves were constructed, and differences between the survival curves were evaluated using the log-rank (Mantel–Cox) test. This method allowed us to compare the survival distributions of two or more groups, providing insights into the effectiveness of different treatments over time. Furthermore, to estimate the risk (hazard ratios) associated with the different treatments and control conditions, Cox proportional hazards regression analysis was employed. This analysis facilitated the examination of the impact of multiple variables on survival time simultaneously, offering a comprehensive understanding of factors influencing survival rates within the study population.

## 3. Results

### 3.1. Co-Culture of L. plantarum, L. acidophilus, and A. kunkeei

**Figure 1:**
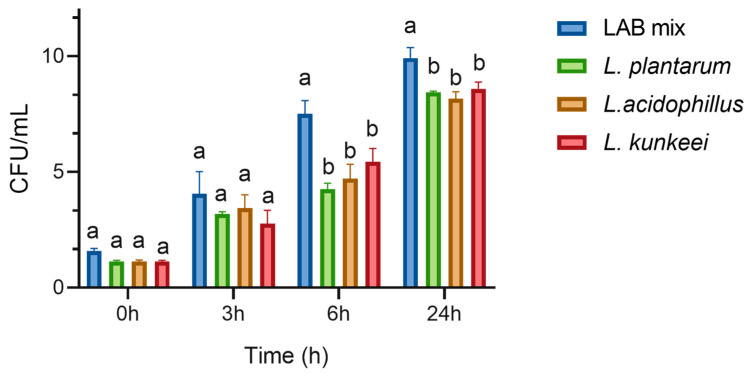
Growth of *L. plantarum*, *A. kunkeei*, and *L. acidophilus* is independent and in co-culture (LAB mix). Values are represented as the means of three independent determinations ± standard deviation. Lowercase letters indicate significant differences among the evaluated LABs at different time intervals (*p* < 0.05).

A co-culture is a straightforward model to examine interactions among different species and is crucial for designing a probiotic product, as strains can affect each other’s growth. This setup simulates in vivo environments where microorganisms interact, exchanging chemical signals and metabolites. Figure 1 shows the results obtained from the growth in co-culture of *L. plantarum*, *L. acidophilus*, and *A. kunkeei*. Analyzing the co-culture of *L. plantarum*, *L. acidophilus*, and *A. kunkeei* and the growth of each strain independently, it can be observed that in the first hours of cultivation (initial moment—time 0 and after 3 h), no significant differences are observed between the samples. After six hours, significant differences were observed between bacterial growth individually and in co-culture with other strains. These differences persisted even after 24 h.

The co-culture displayed notable synergistic interactions, especially in the mid- to late-log phase, where a significant biomass increase was observed. This suggests that the metabolic by-products of one species may serve as nutrients or growth stimulants for others, enhancing overall productivity. The results indicate minimal competitive exclusion among the strains. *L. plantarum*, *L. acidophilus*, and *A. kunkeei* coexisted without significant competition, suggesting balanced ecological niche partitioning.

### 3.2. Mutual Antagonistic Activity between Tested LAB Strains

The primary aim of this study was to assess the potential for mutual antagonism among the tested LAB strains to determine their compatibility for inclusion in a probiotic bioformulation. The results indicated that no zones of growth inhibition were observed in any of the investigated LAB strains, demonstrating that none exhibited mutual antagonistic activity. The lack of growth inhibition zones suggests that these LAB strains do not produce compounds that inhibit each other’s growth, which is essential for multi-strain probiotic formulations.

### 3.3. Osmotic Stress Resistance Test—LAB Mix Capacity to Survive in Syrups

**Figure 2:**
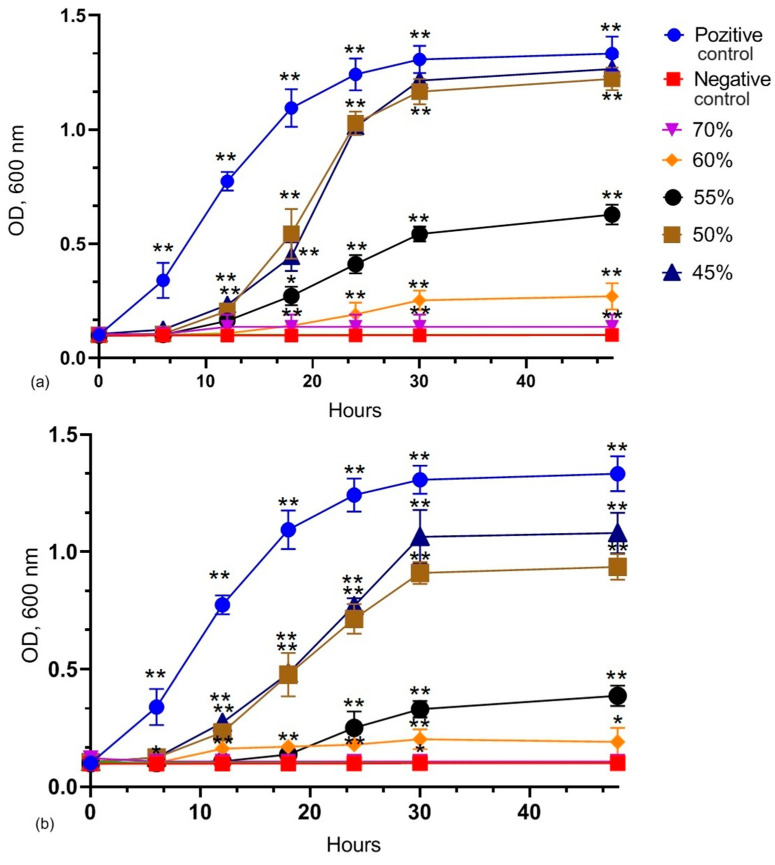
The effect of different syrup concentrations on LAB mix development. (**a**) Development of LAB mix in different concentrations of sugar syrup; (**b**) development of LAB mix in different concentrations of glucose + fructose syrup. For both graphs, the positive control is represented by the MRS medium with LAB-mixed bacteria, and the negative control is represented by the MRS medium without bacteria. Values are represented as the mean of three independent determinations ± standard deviation, highlighting the variability in the LAB response. Asterisks indicate significant differences between the development rate of LABs in different concentrations of syrups (* *p* < 0.05; ** *p* < 0.001).

Figure 2 shows the dynamics of bacterial development during 48 h in two syrups of different concentrations. It can be observed that syrup samples with concentrations higher than 55% are not favorable for bacterial development. The line representing the 80% carbohydrate concentration is not visible in the graph as it is completely overlapped by the negative control line, indicating that bacteria did not grow at this concentration. The graph illustrates that the lag phase has a longer duration in the sugar syrup samples compared to the positive control, which represents the natural growth curve of bacteria, indicating that LAB requires a period to adapt to osmotic stress before entering the exponential growth phase. The stationary phase follows: due to resource depletion or the accumulation of metabolic by-products, the bacterial growth rate slows down and reaches equilibrium. The number of viable cells remains constant, as the rate of cell division equals the rate of cell death.

**Figure 3:**
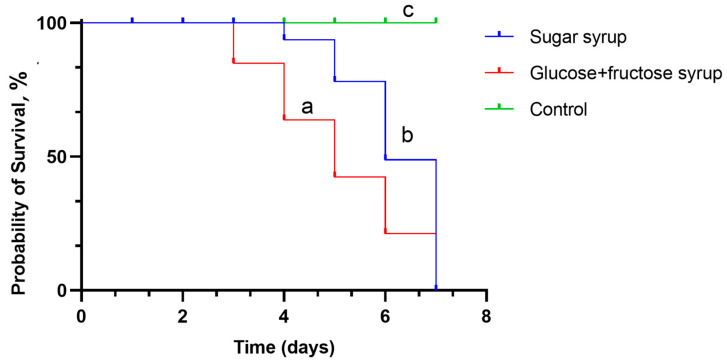
The Kaplan–Meier survival curve of LAB mix in syrup over a period of 7 days. Sugar syrup = survival rate of LAB in sugar syrup (blue line). Glucose + fructose syrup = survival rate of LAB in glucose + fructose syrup. Control = LAB without syrup, with MRS medium. Curves labeled with different letters are significantly different (*p* < 0.05).

Additionally, the survival rate of lactobacilli was tested in both sugar syrup and glucose and fructose syrup, both having a concentration of 50%, for 7 days. The results (see Figure 3) indicate that lactobacilli demonstrate a significantly prolonged and higher survival rate when exposed to sugar syrup than glucose and fructose syrup. The vitality of lactobacilli cells in both types of syrups was observed during this experiment, and on the third day of incubation, there was a noticeable decline in the number of viable cells in the fructose and glucose syrups. On the other hand, lactobacilli that were exposed to sugar syrup continued to have a high and steady cell density over the full seven-day investigation.

After examining the survival rate graphs of LAB in sugar syrup and glucose + fructose syrup over 7 days, a clear trend favoring sugar syrup as a medium for supporting LAB viability is observed. The Kaplan–Meier analysis was used to estimate survival functions for each treatment group. The survival analysis, as determined by the log-rank (Mantel–Cox) test, indicated a chi-square value of 26.19 with 2 degrees of freedom, yielding a *p*-value of less than 0.01. This result signifies statistically significant differences in the survival curves of the LAB strains across the treatment groups.

Furthermore, hazard ratios (HR) were calculated to assess the relative risk of mortality among the bacterial strains subjected to syrup treatments compared to the control group, in which only LAB strains and MRS culture medium were used. For the strains treated with sugar syrup, an HR of 0.75 (95% CI = 0.55–1.02) was observed, suggesting a 25% decrease in the relative risk of mortality. This may indicate a beneficial effect of sugar syrup on the viability of LAB strains, although the confidence interval marginally includes the null value, indicating that results should be interpreted with caution.

Conversely, the glucose + fructose syrup treatment group exhibited an HR of 1.21 (95% CI = 0.89–1.76), indicating a 21% increase in the relative risk of mortality. This raises concerns about the potential adverse effects of glucose + fructose syrup on the survival of LAB strains, though the confidence interval indicates that further research is necessary to confirm these findings.

### 3.4. LAB Strains’ Capacity to Survive in Simulated In Vitro Gastrointestinal Digestive Conditions

**Figure 4:**
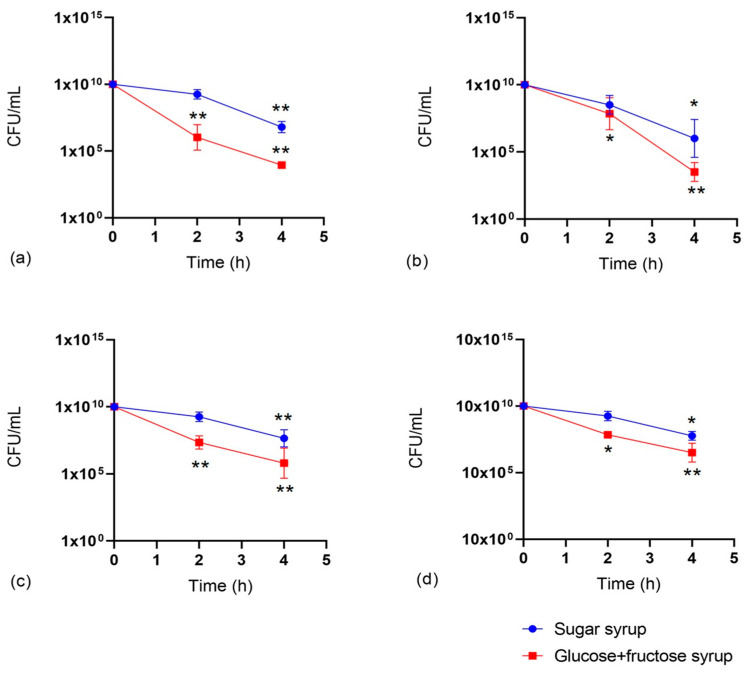
Survival rate of (**a**) *L. plantarum*, (**b**) *L. acidophilus*, (**c**) *A. kunkeei*, and (**d**) LAB mix in simulated in vitro gastric (t < 2 h) and intestinal (t > 2 h) conditions. The blue line (spherical markers) indicates LAB mixes with sugar syrup, and the red line (square markers) indicates LAB mixes with glucose and fructose syrup incubated with gastric and intestinal juices. Values are represented as the mean of three independent determinations ± standard deviation. Asterisks indicate significant differences between the survival rate of LABs in gastric and intestinal conditions based on the syrup mixtures (* *p* < 0.05; ** *p* < 0.001).

The resistance of LAB and LAB mix to the action of digestive juices was tested to confirm that the three LABs could withstand the challenging conditions of the bee’s digestive system, and the results are illustrated in Figure 4.

LAB and the LAB mix exhibit a significant decrease in survival rates under both simulated gastric (t < 2 h) and intestinal (t > 2 h) conditions. In all cases, bacteria incubated with glucose and fructose syrup showed a more rapid and significant decline in viability compared to those incubated with sugar syrup. The statistical differences (* *p* < 0.05; ** *p* < 0.001) indicate that bacteria maintain greater resistance and viability in the presence of sugar syrup under both gastric and intestinal conditions. Specifically, *L. plantarum* (*p* < 0.01) and *L. acidophilus* (*p* < 0.05) demonstrated a more pronounced decrease in survival compared to *A. kunkeei* (*p* < 0.01) and the LAB mix (*p* < 0.01). This highlights their acid tolerance, a critical probiotic trait, allowing them to withstand pepsin, α-amylase, and acidic gastric conditions. In the intestinal phase, increased LAB mortality (*p* < 0.01) was noted, likely due to pancreatin and bile salts. Nevertheless, all strains maintained a cell count exceeding 10^4^ CFU/mL, above the known threshold of 10^3^ CFU/mL for probiotic resistance to bile salts. This indicates that these LABs can survive in conditions like honeybee gastrointestinal tracts.

### 3.5. Biofilm Formation

**Figure 5:**
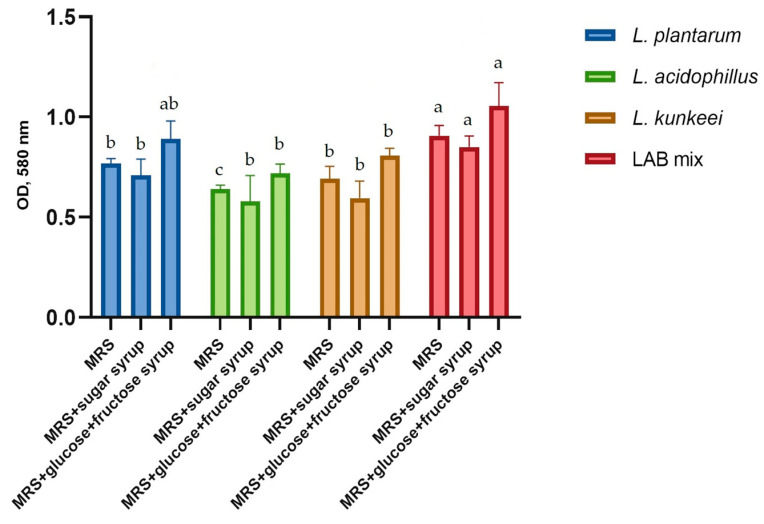
Comparison of capacities for biofilm formation by tested LAB. Influence of MRS medium supplementation with sucrose, glucose, and fructose syrups on biofilm formation. Values represent the mean of six biological repeats ± standard deviation. Different letters indicate statistically significant differences between groups (*p* < 0.05).

Figure 5 illustrates the effect of sucrose, glucose, and fructose on biofilm formation by LAB and their LAB mixture. In order to study the influence of sugars on biofilm formation, MRS medium was used with and without supplements with 5% sucrose syrup, glucose, and fructose syrup, respectively.

LAB mix exhibited the highest biofilm production capacity, closely followed by *L. plantarum*. In all tested conditions, including MRS, MRS supplemented with sugar syrup, and MRS supplemented with glucose + fructose syrup, the LAB mix showed the highest optical density (OD) values at 580 nm, indicating superior biofilm formation compared to the individual strains. The highest biofilm formation values for all LAB strains are observed in the MRS + glucose + fructose syrup condition. This condition consistently resulted in significantly higher biofilm production compared to the MRS medium and MRS + sugar syrup conditions. Different letters indicate statistically significant differences between groups, showing that MRS + glucose + fructose syrup significantly enhances biofilm formation for all tested strains. This enhancement is particularly evident in comparison to the other conditions, which generally showed lower and comparable biofilm formation levels. This indicates that the specific composition of the medium plays an important role in the biofilm-forming capacity of these bacteria, with glucose and fructose providing a more favorable environment for biofilm development.

### 3.6. Auto-Aggregation Test

All strains exhibited an increasing trend in aggregation as the incubation time extended, highlighting a time-dependent enhancement in their ability to form aggregates. The results of the auto-aggregation test are presented in Table 1.

*L. plantarum* begins with a minimal aggregation of 3.45% at 1 h, which significantly escalates to 66.81% by 24 h. Similarly, both *L. acidophilus* and *A. kunkeei* start below 4% but exceed 68% aggregation after 24 h. The LAB mix demonstrates the strongest aggregation, beginning at 3.92% and reaching 71.91% at 24 h, highlighting the enhanced cohesive properties when multiple strains are combined. Over time, all strains exhibit an increase in auto-aggregation, with the LAB mix demonstrating the greatest capacity for auto-aggregation after 24 h. Statistical differences indicate that the LAB mix has significantly better auto-aggregation ability compared to the individual strains, highlighting its potential robustness in biofilm formation and stability.

### 3.7. Lactic Acid

The lactic acid production profile of the LAB strains shows significant differences between the analyzed LAB and their mixt. *L. plantarum* produced the highest amount of lactic acid, significantly more than *L. acidophilus* and *A. kunkeei*. The lactic acid content is presented in Table 2. The LAB mix yielded 10.16 ± 0.64 g/L, indicating that co-culturing these strains resulted in lower combined lactic acid production compared to *L. plantarum* alone. These findings suggest that while *L. plantarum* is the most prolific producer of lactic acid, interactions in the LAB mix may reduce overall lactic acid output.

### 3.8. Antioxidant Activity

The antioxidant activities of *L. plantarum*, *A. kunkeei*, *L. acidophilus*, and an LAB mix were assessed using DPPH, ABTS, and FRAP assays. *L. plantarum* shows relatively high antioxidant activity, particularly in the DPPH method, but is lower in FRAP compared to *L. acidophilus*. *L. acidophilus* demonstrates good antioxidant activity, especially in the ABTS and FRAP methods. *A. kunkeei* has the lowest antioxidant activity across all methods, indicating its relatively weaker antioxidant potential. The LAB mix consistently exhibits the highest antioxidant activity across all three methods (DPPH, ABTS, and FRAP), indicating strong antioxidant potential. The results are presented below in Table 3.

These results suggest that the combined LAB strains in the mix may synergize to enhance overall antioxidant potential more effectively than any single strain. This enhancement might be attributed to varied metabolic interactions and complementary antioxidative mechanisms within the mix, pointing to the potential advantages of multi-strain formulations in probiotic products aimed at reducing oxidative stress.

### 3.9. Antimicrobial Activity

#### 3.9.1. Disk Diffusion Method

The antimicrobial activity of LAB and LAB mix against various pathogenic strains, as reflected by the inhibition diameters, provides a nuanced understanding of their potential as biocontrol agents. In general, all probiotic strains had antimicrobial effects on the tested bacteria, but the results highlight significant differences in inhibition efficacy, with the LAB mix generally showing superior performance compared to the individual strains (see Table 4).

Among the individual strains, *L. plantarum* showed the best inhibitory effect. The LAB mix consistently demonstrated superior inhibition across all tested strains, benefiting from synergistic interactions. Notably, it improved the inhibition of bee pathogens like *P. larvae* and *M. plutonius*, with inhibition zones of 17.56 ± 0.65 mm and 18.90 ± 0.83 mm, respectively, surpassing any single strain. This suggests a potential role for LAB mixtures in preventing foulbrood diseases in apiculture.

Additionally, the LAB mix performed well against common foodborne and clinical pathogens such as *S. aureus*, *B. cereus*, and *E. coli*, indicating its potential for food safety applications. However, its effectiveness against *P. aeruginosa* remains limited, highlighting the pathogen’s resilience. Regarding the antifungal activity, the probiotic strains showed inhibition diameters between 5.44 ± 0.25 and 6.14 ± 0.83 mm, and the best result was observed for the LAB mix, which was 7.95 ± 0.50 mm.

#### 3.9.2. Microplate Assay

**Figure 6:**
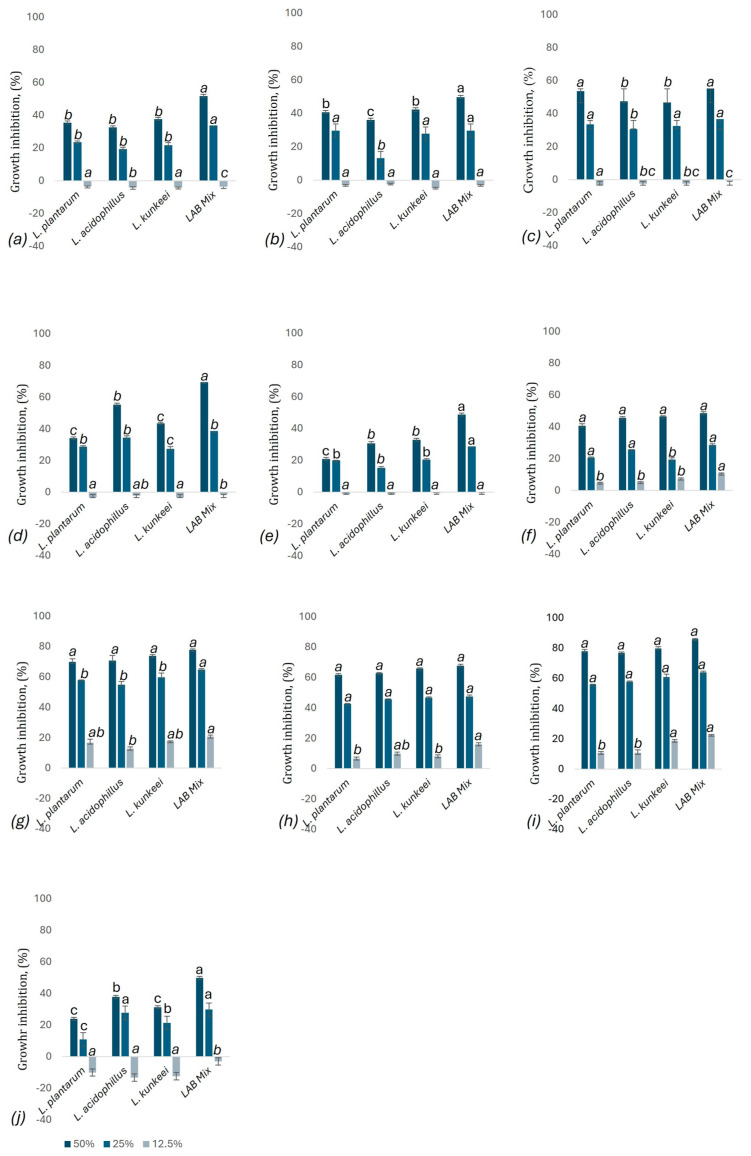
Growth inhibition (%) of pathogenic strains: (**a**) *S. aureus*, (**b**) *B. cereus*, (**c**) *S. enteritidis*, (**d**) *E. coli*, (**e**) *P. aeruginosa*, (**f**) *E. faecalis*, (**g**) *M. plutonius*, (**h**) *P. larvae*, (**i**) *P. alvei*, (**j**) *C. albicans* by LAB supernatants at physiological pH of LAB evaluated by microplate method. The dark blue on the graphic represents the concentration of 50% of LAB supernatants, the blue color represents the concentration of 25% of LAB supernatants, and the light blue color represents the concentration of 12.5% of LAB supernatants. Values are represented as the mean of three independent determinations ± standard deviation. Different letters between the same LAB supernatant concentration denote significant differences (*p* < 0.05).

The analysis of growth inhibition (Figure 6) of various pathogenic strains by LAB supernatants at different concentrations (50%, 25%, and 12.5%) reveals that the highest concentration of 50% consistently demonstrates the greatest inhibitory effect across all LAB strains and pathogens. This trend is evident in all graphs (a–j), where the dark blue bars (50% concentration) show significantly higher growth inhibition compared to the lower concentrations (25% and 12.5%). This statistical significance, indicated by different letters, highlights that the 50% concentration is markedly more effective (*p* < 0.05) than the lower concentrations, reinforcing the conclusion that higher concentrations of LAB supernatants are more potent in inhibiting pathogenic growth. *S. aureus* emerges as the most sensitive bacterium to the LAB supernatants, particularly to the LAB mix at 50% concentration, which shows the highest growth inhibition. Similarly, *M. plutonius* and *P. larvae* also exhibit high sensitivity, with the LAB mix at 50% concentration demonstrating significant inhibitory effects; instead, *P. aeruginosa* was the most resistant pathogen, showing minimal to no inhibition across all LAB strains and concentrations. Even the LAB mix, which generally showed the highest inhibition, had a relatively low effect on *P. aeruginosa*. The LAB mix consistently demonstrated the highest inhibition percentages across all tested pathogens and concentrations, indicating a strong synergistic effect. This suggests that the combination of LAB strains enhances overall antimicrobial activity, while *L. acidophilus* was generally the least effective individual strain, showing lower inhibition percentages compared to *L. plantarum* and *A. kunkeei*. Among the individual strains, *A. kunkeei* generally exhibited higher inhibition, especially against *P. larvae*, *P. alvei*, and *M. plutonius*, whereas *L. acidophilus* was the least effective.

### 3.10. Antibiotic Susceptibility

The antibiotic susceptibility profile of the tested LAB is presented in Table 5.

The results reveal distinct resistance patterns, reflecting intrinsic and maybe acquired resistance mechanisms. All strains are intrinsically resistant to aminoglycosides such as streptomycin and kanamycin but show variability with gentamicin; *L. plantarum* and LAB mix are moderately susceptible, while *L. acidophilus* and *A. kunkeei* are susceptible. All strains are susceptible to chloramphenicol but exhibit intrinsic resistance to vancomycin, indicating common resistance mechanisms inherent to LAB. *L. plantarum* and *L. acidophilus* are susceptible to lincomycin, whereas *A. kunkeei* and LAB mix are resistant. For macrolides, *L. plantarum* and *L. acidophilus* are susceptible to erythromycin and tylosin, while *A. kunkeei* and LAB mix exhibit intrinsic resistance. All strains show intrinsic resistance to ampicillin and sulfonamides and moderate susceptibility to tetracyclines. *A. kunkeei* shows unique susceptibility to gentamicin compared to other strains, which exhibit resistance or moderate susceptibility.

## 4. Discussion

Probiotics are increasingly used in bee treatment, but it is essential to test bacterial combinations to ensure they enhance rather than antagonize each other’s activity. This study evaluated the potential of a mixture of three bacterial strains—*L. plantarum*, *L. acidophilus*, and *A. kunkeei*—for use in a probiotic product for honeybees.

### 4.1. Interactions and Compatibility among Tested LAB

In this study, the interactions and compatibility among three probiotic LAB strains were investigated: *L. plantarum* ATCC 8014, *L. acidophilus* ATTC 700396, and *A. kunkeei* ATCC 700308. The results indicate that co-culturing these strains demonstrates potential synergy; this is a promising finding as it suggests that these strains can coexist without inhibiting each other’s growth, which is crucial for developing a stable and effective probiotic product. The combined use of these strains may offer broader probiotic benefits, including improved survival during storage and gastrointestinal transit and increased stress resistance [34,61,62]. Co-culturing these strains has been reported to enhance growth and metabolic activity, producing beneficial metabolites such as lactic acid and short-chain fatty acids, which inhibit pathogenic bacteria and support a balanced gut microbiota [63,64]. Additionally, co-culturing improves resistance to environmental stresses like acidic conditions [65] and bile salts, which are common in the gastrointestinal tract, benefiting the development of robust probiotic formulations [65,66]. *L. plantarum* and *L. acidophilus* together stimulate beneficial metabolites such as lactic acid and short-chain fatty acids, which are crucial for gut health [67]. Previous studies have shown that the co-culture of *L. plantarum* enhances digestive and immune support [68]. *A. kunkeei* improves gut microbiota and inhibits pathogens [69], and *L. acidophilus* maintains balanced intestinal flora and provides infection protection [70]. The absence of antagonistic interactions among these strains is promising for their inclusion in a honeybee probiotic supplement, fostering a stable and effective multi-strain product. Few studies have investigated the antagonistic effects of LAB strains intended for probiotics in bees [49,71]. This study supports the use of LAB combinations to enhance honeybee health and resilience. The lack of antagonism allows potential synergistic effects, improving gut colonization [72], immune modulation, and superior health benefits compared to single-strain probiotics, an aspect also reported by other authors [73].

### 4.2. Survival and Resistance under Various Conditions

Probiotic LAB candidates for honeybees must be resistant to high carbohydrate concentrations, as they are often delivered through sugar syrup [44,74,75]. In this study, lactobacilli maintained high cell density in sugar syrup over seven days, compared to glucose and fructose syrups. This aligns with previous findings [76,77], which suggest that lactobacilli may benefit from osmotic protection in the presence of sucrose, thereby contributing to better survival and highlighting the impact of syrup composition on the survival rates of LAB strains. Additionally, Iorizzo et al. [78] noted that *A. kunkeei* strains isolated from the digestive tract of honeybees showed tolerance to high sugar concentrations, while Leska et al. [49] tested the viability of ten isolated LAB strains and one reference strain, *A. kunkeei* DSM 12361, in syrups of varying concentrations of glucose, fructose, or sucrose, and the reported results showed a better survival rate of LAB in the syrup with glucose and fructose without sucrose. In this study, the best survival rates for LAB were observed in syrups with carbohydrate concentrations below 55% for both tested syrups, suggesting commercial syrups might need dilution before being used to administer the probiotic mix. This highlights the need for syrups that allow easy concentration adjustment to meet the needs of both bees and probiotics, supporting bee health and daily activities [75,79].

Another critical aspect is probiotics’ ability to withstand digestive enzymes, stomach acidity, and bile salts, which are crucial for colonizing the honeybee digestive tract [45]. This study found that *L. plantarum*, *L. acidophilus*, *L. kunkeei*, and their mixture survived in vitro simulated gastrointestinal conditions despite a decrease in total bacteria. Leska et al. [49] reported high survival rates for most LAB strains, though mortality increased in simulated intestinal juice due to pancreatin and bile salts. Similarly, Tokatli et al. [80] observed that *L. brevis* and *L. plantarum* survived better in gastric juice than in intestinal conditions, showing strain-dependent resistance to bile salts. Additionally, Mantzourani et al. [81] also highlighted strain-dependent variations in survival rates. These findings emphasize the need for probiotics resistant to gastric and intestinal juices for effective formulations, necessitating comprehensive in vitro evaluations [41,45,82].

Following their ability to withstand digestive stresses, another vital consideration is the resistance of LAB strains to antibiotics commonly used in veterinary medicine, and this resistance is crucial for their deployment in environments where these antibiotics may be present [83]. LAB mix exhibits a broad resistance profile, being generally resistant to most antibiotics except chloramphenicol, with moderate susceptibility to tetracyclines. *L. plantarum* and *L. acidophilus* display similar resistance patterns but with some additional susceptibilities. *A. kunkeei* was broadly susceptible but resistant to lincosamides and macrolides. The resistance of LAB strains to antibiotics, in this case, is typically intrinsic, meaning it is naturally occurring due to inherent mechanisms within their cell wall structure and not acquired through gene transfer [84]. Similar antibiotic susceptibility patterns for *L. plantarum* and *L. acidophilus* have been reported by other authors [85,86,87]. Similarly, the antibiogram profile of *L. kunkeei* also shows high resistance to several antibiotics [88]. These antibiotic susceptibility patterns underscore the need for careful selection and management of LAB strains in probiotic products designed for bees to enhance their health and resilience against pathogens while considering the impact of antibiotic use in apiculture and agriculture.

### 4.3. Biofilm Formation, Auto-Aggregation, Lactic Acid Production and Antioxidant Activity

In this study, the supplementation of medium with glucose and fructose syrup significantly enhanced biofilm formation by LAB. This aligns with the study of Cai et al. [89] and Isom et al. [90], showing carbohydrate availability boosts biofilm production. The ability of probiotic strains to form biofilms is a critical factor for their colonization and persistence in the gut and also facilitates close interactions between probiotic strains [91]. The results have shown that co-culturing LABs can enhance biofilm formation, providing a protective environment that supports their survival and activity in the gut, as also reported in the literature [92].

Regarding auto-aggregation, results showed an increase over time, consistent with findings from other studies [93]; this likely involves cellular adhesion molecules and changes in cell surface properties, enhancing stability. Furthermore, Chen et al. [94] reported auto-aggregation percentages for LAB strains ranging from 10.75% to 17.62% after 5 h, which is consistent with our findings. Aggregation enhances survival in acidic and bile environments, aids intestinal colonization, modulates gut microbiota, and exerts antimicrobial effects [95,96,97]. It also promotes a balanced immune response, reducing inflammation and improving stability during processing and storage, increasing supplement efficacy [95].

*L. plantarum* shows moderate DPPH and ABTS activity but lower FRAP; *L. acidophilus* excels in ABTS; and *A. kunkeei* exhibits the least activity in all assays. The LAB mix outperforms individual strains in all assays, demonstrating enhanced overall antioxidant capacity. This increased activity may result from varied metabolic interactions between strains, boosting the production or stability of antioxidant compounds [98]. Other studies also note variances in antioxidant activities among LAB strains like *L. plantarum*, *A. kunkeei*, and *L. acidophilus* [11,70,99]. The obtained results are similar to those in the literature; *L. plantarum* is strong at scavenging free radicals and reducing oxidative stress [98]. *L. acidophilus* shows good potential, particularly in reducing power assays [100], and *A. kunkeei*, while beneficial, has lower antioxidant activity [101]. These findings emphasize the value of multi-strain LAB formulations in enhancing antioxidant activity, which may facilitate the development of natural biocontrol strategies against pathogens, reduce dependence on chemical antibiotics, and promote sustainable practices [34].

*L. plantarum* produced the highest lactic acid levels, more than *L. acidophilus* and *A. kunkeei*. However, the LAB mix showed lower lactic acid production than *L. plantarum* alone, suggesting that inter-strain interactions in co-culture may reduce overall lactic acid output. This highlights the complexity of microbial dynamics in mixed cultures, where competitive or inhibitory interactions can impact metabolic productivity [65]. Lactic acid inhibits pathogenic bacteria by lowering pH; lower lactic acid output in mixed cultures might reduce antimicrobial efficacy, but multiple strains might introduce other antimicrobial mechanisms [63], such as bacteriocin production.

### 4.4. Antimicrobial Activity

The LAB mix showed the highest inhibition diameters and growth inhibition, particularly notable against bee pathogens like *S. aureus*, *P. larvae*, and *M. plutonius*. Individual strains also exhibited significant activity, with *A. kunkeei* showing higher efficacy against bee pathogens than *L. plantarum* and *L. acidophilus*. The mix’s enhanced performance suggests synergistic interactions, which are crucial for developing effective probiotics. The results obtained are similar to those reported by Danilova et al. [102], who investigated the antimicrobial activity of *L. plantarum* against *E. coli*, *P. aeruginosa*, *S. pyogenes*, and *S. aureus*. Although the 48 h culture supernatant showed inhibitory effects on both Gram-positive and Gram-negative pathogens, *P. aeruginosa* was the most resistant. Other authors [103] reported that LAB strain metabolites, including *L. plantarum*, *L. acidophilus*, *L. rhamnosus*, *L. apis*, and *P. acidilactici*, were tested against *M. plutonius*, and the results showed high inhibition of the pathogen. Moreover, the mixture of the four most potent isolates within 96 h reduced *M. plutonius* survival to 0%.

Furthermore, Iorizzoet et al. [104] tested four *L. plantarum* strains and four *A. kunkeei* strains isolated from the gastrointestinal tract of *Apis mellifera* L. and highlighted their in vitro inhibitory activity against the vegetative growth of *P. larvae* and *M. plutonius*. Another study [105] reported that a probiotic mixture based on *A. kunkeei* reduced the mortality associated with *P. larvae* in honeybee larvae and decreased the number of *Nosema ceranae* spores in adult bees. Other studies [34] have shown the antibacterial effect of *L. kunkeei* in vivo on bees infested with *P. larvae*. Regarding foodborne pathogens, studies [106] showed that lactobacilli isolated from honey, among which *L. plantarum*, *L. kunkeei*, *L. rhamnosus*, and others, showed good antimicrobial activity against *S. aureus*, *S. enteritidis*, *E. coli*, and *B. cereus*, with the highest percent inhibition observed against *E. coli* O157 H7. It is known that lactobacilli generate antimicrobial effects by producing organic acids (lactic and acetic acids) that lower pH [71], secreting bacteriocins, producing hydrogen peroxide, competing for nutrients and adhesion sites, and generating short-chain fatty acids like butyric acid [26,72,107]. These mechanisms collectively create an inhospitable environment for pathogens and inhibit their growth [51,108].

## 5. Conclusions

This study demonstrates the potential of three LAB strains—*L. plantarum*, *L. acidophilus*, and *A. kunkeei*—to be put in the same formula as probiotics for enhancing bee health. The individual and combined effects of these strains, referred to as the LAB mix, were comprehensively evaluated through a series of in vitro tests. The LAB strains showed no mutual antagonistic activity and significant resilience under high osmotic stress and simulated gastrointestinal conditions. The LAB mix exhibited superior biofilm formation, antioxidant activity, and antimicrobial efficacy against key bee pathogens. These results suggest that a multi-strain probiotic formulation could play a critical role in improving bee health and mitigating the impact of pathogens and environmental stressors. Future research should focus on conducting in vivo studies to validate the LAB mix’s impact on colony strength, pathogen resistance, and productivity in real-world settings. Additionally, investigating optimal delivery methods such as sugar syrups and pollen patties is crucial to maximizing probiotic viability and efficacy. Determining the optimal dosages and concentrations for significant health benefits in honeybees is also essential. Long-term studies are needed to assess the effects of LAB mix use on bee health and hive productivity over multiple seasons. Finally, detailed microbiome analyses before and after probiotic treatment will help understand the changes in the bee gut microbiota and their contribution to improved health outcomes.

## Figures and Tables

**Table 1:** Percentages of auto-aggregation (%) of tested LAB evaluated after 1 h, 2 h, 6 h, and 24 h of incubation.

Strains	1 h	2 h	6 h	24 h
*L. plantarum*	3.45 ± 0.12 ^a^	11.51 ± 2.64 ^b^	20.15 ± 1.12 ^c^	66.81 ± 2.11 ^b^
*L. acidophilus*	2.81 ± 0.17 ^b^	15.02 ± 1.29 ^a^	24.09 ± 0.65 ^a,b^	68.13 ± 3.02 ^b^
*A. kunkeei*	3.67 ± 0.32 ^a^	15.82 ± 1.32 ^a^	23.71 ± 0.98 ^b^	68.00 ± 1.46 ^b^
LAB mix	3.92 ± 0.18 ^a^	16.78 ± 0.95 ^a^	25.54 ± 1.17 ^a^	71.91 ± 1.00 ^a^

Results represent the mean of three independent determinations ± standard deviation. Within the same column, different letters indicate significant differences (*p* < 0.05).

**Table 2:** Organic acid (g/L) profile of LAB strains.

Organic Acids	*L. plantarum*	*L. acidophilus*	*A. kunkeei*	LAB Mix
Lactic acid	17.42 ± 0.39 ^a^	10.34 ± 0.25 ^b^	4.17 ± 0.22 ^c^	10.16 ± 0.64 ^b^

Values are represented as the mean of three independent determinations ± standard deviation. Within the same row, different letters indicate significant differences (*p* < 0.05).

**Table 3:** Antioxidant activity of LAB strains.

Bacteria	DPPH Method, %	ABTS Method, %	FRAP Method, µmol Trolox/mL
*L. plantarum*	49.33 ± 0.21 ^b^	46.89 ± 0.34 ^b^	42.21 ± 0.24 ^b^
*L. acidophilus*	42.14 ± 0.32 ^c^	54.73 ± 0.45 ^a^	45.35 ± 0.31 ^b^
*A. kunkeei*	31.71 ± 0.28 ^d^	47.95 ± 0.76 ^b^	32.11 ± 0.28 ^c^
LAB mix	55.05 ± 0.27 ^a^	57.51 ± 0.38 ^a^	61.23 ± 0.14 ^a^

Values are represented as the mean of three independent determinations ± standard deviation. Within the same column, different letters indicate significant differences (*p* < 0.05).

**Table 4:** Inhibition diameters (mm) of LAB against tested pathogenic strains.

Family	Genus and Species	*L. plantarum*	*L. acidophilus*	*A. kunkeei*	Lab MIX
Bacillaceae	*Bacillus cereus*	5.67 ± 0.11 ^b^	5.23 ± 0.50 ^b^	4.89 ± 0.38 ^c^	6.95 ± 0.25 ^a^
Enterobacteriaceae	*Escherichia coli*	4.25 ± 0.79 ^b^	3.70 ± 0.18 ^b^	4.00 ± 0.53 ^b^	7.34 ± 0.25 ^a^
	*Salmonella enteritidis*	8.25 ± 0.61 ^a^	7.34 ± 0.23 ^b^	7.52 ± 0.16 ^b^	9.50 ± 0.10 ^a^
Enterococcaceae	*Enterococcus faecalis*	3.15 ± 0.52 ^b^	2.00 ± 0.12 ^c^	3.00 ± 0.53 ^b^	5.14 ± 0.40 ^a^
	*Melissococcus plutonius*	13.00 ± 0.58 ^b,c^	12.16 ± 0.74 ^c^	14.25 ± 0.21 ^b^	18.90 ± 0.83 ^a^
Paenibacillaceae	*Paenibacillus alvei*	11.50 ± 0.65 ^b^	11.50 ± 0.46 ^b^	13.50 ± 0.80 ^b^	16.25 ± 0.50 ^a^
	*Paenibacillus larvae*	10.34 ± 0.80 ^c^	11.91 ± 0.73 ^b,c^	12.04 ± 0.59 ^b^	17.56 ± 0.65 ^a^
Pseudomonadaceae	*Pseudomonas aeruginosa*	0.50 ± 0.10 ^b^	0.00 ± 0.00 ^c^	0.00 ± 0.00 ^c^	1.25 ± 0.15 ^a^
Staphylococcaceae	*Staphylococcus aureus*	6.78 ± 0.11 ^b^	6.47 ± 0.13 ^b^	5.72 ± 0.24 ^c^	8.00 ± 0.56 ^a^
Saccharomycetaceae	*Candida albicans*	6.14 ± 0.83 ^b^	5.44 ± 0.25 ^b^	5.97 ± 0.59 ^b^	7.95 ± 0.50 ^a^

Values are represented as the mean of three independent determinations ± standard deviation. Within the same row, different letters indicate significant differences (*p* < 0.05).

**Table 5:** Antibiotic susceptibility of LAB.

Antibiotic Class	Antibiotic (Concentration)	*L. plantarum*	*L. acidophilus*	*A. kunkeei*	LAB Mix
Aminoglicozide	Streptomycin (25 µg)	R	R	R	R
	Kanamycin (30 µg)	R	R	R	R
	Gentamicin (30 µg)	MS	R	S	R
Amfenicoli	Chloramphenicol (50 µg)	S	S	S	S
Glicopeptide	Vancomycin (30 µg)	R	R	R	R
Lincosamide	Lincomycin (15 µg)	R	R	S	R
Macrolide	Erythromycin (30 µg)	S	S	S	S
	Tylosin (30 µg)	R	R	S	R
Beta-lactamine	Ampicillin (10 µg)	R	S	R	R
Sulfonamide	Sulfonamides (300 µg)	R	R	R	R
Tetracicline	Tetracycline (30 µg)	MS	MS	MS	MS
	Oxytetracycline (30 µg)	MS	MS	MS	MS

R: resistant, MS: moderately susceptible, S: susceptible. The results are represented as the mean of three independent determinations.

## Data Availability

The original contributions presented in the study are included in the article/Supplementary Materials, further inquiries can be directed to the corresponding author.

## Supplementary Materials

The following supporting information can be downloaded at: https://www.mdpi.com/article/10.3390/microorganisms12061249/s1, Table S1: Growth of *L. plantarum*, *A. kunkeei*, and *L. acidophilus* independent and in co-culture (LAB mix); Table S2: The effect of different syrup concentrations on LAB mix development; Table S3: Resistance of tested LAB strains in simulated in vitro gastric (t < 2 h) and intestinal (t > 2 h) conditions; Table S4: Comparison of capacities of biofilm formation by tested LAB. Influence of MRS medium supplementation with sucrose, glucose, and fructose syrups on biofilm formation; Table S5: Growth inhibition (%) of tested patogenic strains by LAB supernatants at physiological pH of LAB evaluated by microplate method.
