# Supplementary material for: Evaluation of Functional Properties of Some Lactic Acid Bacteria Strains for Probiotic Applications in Apiculture
Source: Microorganisms. 2024 Jun 20;12(6):1249. doi: 10.3390/microorganisms12061249 (PMC11205645; doi:10.3390/microorganisms12061249)
Supplement: Supplementary file 1 [file microorganisms-12-01249-s001.zip › microorganisms-3052300-supplementary.pdf]

Supplementary files

Table S1. Growth of *L. plantarum, A. kunkeei, and L. acidophilus* independent and in co-culture (LAB mix).

LAB mix

One-way ANOVA of your kk=4 independent treatments:

source	sum of squares SS	degrees of freedom νν	mean square MS	F statistic	p-value
treatment	106.6534	3	35.5511	81.0312	2.50E-06
error	3.5099	8	0.4387		
total	110.1633	11			

Tukey HSD results

treatments pair	Tukey HSD Q statistic	Tukey HSD p-value	Tukey HSD inferfence
A vs B	6.0117	0.011944	* p<0.05
A vs C	15.0324	0.001005	** p<0.01
A vs D	20.1092	0.001005	** p<0.01
B vs C	9.0206	0.001005	** p<0.01
B vs D	14.0974	0.001005	** p<0.01
C vs D	5.0768	0.029129	* p<0.05

L. plantarum

source	sum of squares SS	degrees of freedom νν	mean square MS	F statistic	p-value
treatment	85.5971	3	28.5324	326.7741	1.05E-08

error	0.6985	8	0.0873	
total	86.2956	11		

Tukey HSD results

treatments pair	Tukey HSD Q statistic	Tukey HSD p-value	Tukey HSD inferfence
A vs B	11.7193	0.001005	** p<0.01
A vs C	19.7477	0.001005	** p<0.01
A vs D	42.8013	0.001005	** p<0.01
B vs C	8.0284	0.002088	** p<0.01
B vs D	31.0821	0.001005	** p<0.01
C vs D	23.0536	0.001005	** p<0.01

L. kunkeei

source	sum of squares SS	degrees of freedom νν	mean square MS	F statistic	p-value
treatment	76.6599	3	25.5533	96.782	1.26E-06
error	2.1122	8	0.264		
total	78.7722	11			

Tukey HSD results

treatments pair	Tukey HSD Q statistic	Tukey HSD p-value	Tukey HSD inferfence
A vs B	7.8697	0.002375	** p<0.01
A vs C	12.1877	0.001005	** p<0.01
A vs D	23.5766	0.001005	** p<0.01
B vs C	4.318	0.061726	insignificant
B vs D	15.7069	0.001005	** p<0.01
C vs D	11.3888	0.001005	** p<0.01

L. acidophillus

source	sum of squares SS	degrees of freedom νν	mean square MS	F statistic	p-value
treatment	96.7133	3	32.2378	170.981	1.36E-07
error	1.5084	8	0.1885		
total	98.2217	11			

Tukey HSD results

treatments pair	Tukey HSD Q statistic	Tukey HSD p-value	Tukey HSD inferfence
A vs B	6.6495	0.006691	** p<0.01
A vs C	17.3051	0.001005	** p<0.01
A vs D	29.91	0.001005	** p<0.01
B vs C	10.6557	0.001005	** p<0.01
B vs D	23.2605	0.001005	** p<0.01
C vs D	12.6049	0.001005	** p<0.01

0 hours

One-way ANOVA of your kk=4 independent treatments:

source	sum of squares SS	degrees of freedom $\nu\nu$	mean square MS	F statistic	p-value
treatment	0.996	3	0.332	3.984	0.0524
error	0.6667	8	0.0833		
total	1.6627	11			

3 hours

source	sum of squares SS	degrees of freedom $\nu\nu$	mean square MS	F statistic	p-value
treatment	2.7636	3	0.9212	2.347	0.1489
error	3.14	8	0.3925		
total	5.9036	11			

6 hours

source	sum of squares SS	degrees of freedom $\nu\nu$	mean square MS	F statistic	p-value
treatment	17.1984	3	5.7328	16.2939	0.0009
error	2.8147	8	0.3518		
total	20.0131	11			

Tukey HSD results

treatments pair	Tukey HSD Q statistic	Tukey HSD p-value	Tukey HSD inferfence
A vs B	8.8935	0.001067	** $p<0.01$
A vs C	8.1674	0.001867	** $p<0.01$
A vs D	6.063	0.011391	* $p<0.05$
B vs C	0.7261	0.899995	insignificant
B vs D	2.8305	0.263665	insignificant
C vs D	2.1044	0.486729	insignificant

24 hours

source	sum of squares SS	degrees of freedom $\nu\nu$	mean square MS	F statistic	p-value
treatment	3.06	3	1.02	6.7571	0.0139
error	1.2076	8	0.151		
total	4.2677	11			

Tukey HSD results

treatments pair	Tukey HSD Q statistic	Tukey HSD p-value	Tukey HSD inferfence
A vs B	4.6993	0.042229	* $p<0.05$
A vs C	6.062	0.011403	* $p<0.05$
A vs D	3.8241	0.10106	insignificant
B vs C	1.3627	0.754813	insignificant
B vs D	0.8753	0.899995	insignificant
C vs D	2.2379	0.439788	insignificant

Table S2. The effect of different syrup concentrations on LAB mix development

sugar syrup

0 hours

source	sum of squares SS	degrees of freedom ν	mean square MS	F statistic	p-value
treatment	0.0002	7	0	2.212	0.0894
error	0.0002	16	0		
total	0.0003	23			

Tukey HSD results

treatments pair	Tukey HSD Q statistic	Tukey HSD p-value	Tukey HSD inferfence
A vs B	1.4311	0.899995	insignificant
A vs C	0.8944	0.899995	insignificant
A vs D	2.1466	0.764741	insignificant
A vs E	0.1789	0.899995	insignificant
A vs F	1.0733	0.899995	insignificant
A vs G	0.3578	0.899995	insignificant
A vs H	2.6833	0.563884	insignificant
B vs C	0.5367	0.899995	insignificant
B vs D	3.5777	0.250718	insignificant
B vs E	1.61	0.899995	insignificant
B vs F	0.3578	0.899995	insignificant
B vs G	1.7889	0.898643	insignificant
B vs H	4.1144	0.13519	insignificant
C vs D	3.0411	0.428875	insignificant
C vs E	1.0733	0.899995	insignificant

glucose+fructose syrup

0 hours

source	sum of squares SS	degrees of freedom ν	mean square MS	F statistic	p-value
treatment	0.0012	7	0.0002	1.8166	0.1527
error	0.0016	16	0.0001		
total	0.0028	23			

Tukey HSD results

treatments pair	Tukey HSD Q statistic	Tukey HSD p-value	Tukey HSD inferfence
A vs B	0.4684	0.899995	insignificant
A vs C	0.7025	0.899995	insignificant
A vs D	3.7469	0.208105	insignificant
A vs E	0.3513	0.899995	insignificant
A vs F	0.0585	0.899995	insignificant
A vs G	0.8782	0.899995	insignificant
A vs H	0.8782	0.899995	insignificant
B vs C	1.1709	0.899995	insignificant
B vs D	4.2153	0.119919	insignificant
B vs E	0.1171	0.899995	insignificant
B vs F	0.5269	0.899995	insignificant
B vs G	1.3465	0.899995	insignificant
B vs H	1.3465	0.899995	insignificant
C vs D	3.0444	0.42761	insignificant
C vs E	1.0538	0.899995	insignificant

C vs F	0.1789	0.899995	insignificant
C vs G	1.2522	0.899995	insignificant
C vs H	3.5777	0.250718	insignificant
D vs E	1.9677	0.831691	insignificant
D vs F	3.2199	0.362321	insignificant
D vs G	1.7889	0.898643	insignificant
D vs H	0.5367	0.899995	insignificant
E vs F	1.2522	0.899995	insignificant
E vs G	0.1789	0.899995	insignificant
E vs H	2.5044	0.630837	insignificant
F vs G	1.4311	0.899995	insignificant
F vs H	3.7566	0.205837	insignificant
G vs H	2.3255	0.697788	insignificant

C vs F	0.644	0.899995	insignificant
C vs G	0.1756	0.899995	insignificant
C vs H	0.1756	0.899995	insignificant
D vs E	4.0982	0.137889	insignificant
D vs F	3.6884	0.222233	insignificant
D vs G	2.8687	0.494475	insignificant
D vs H	2.8687	0.494475	insignificant
E vs F	0.4098	0.899995	insignificant
E vs G	1.2295	0.899995	insignificant
E vs H	1.2295	0.899995	insignificant
F vs G	0.8196	0.899995	insignificant
F vs H	0.8196	0.899995	insignificant
G vs H	0	0.899995	insignificant

6 hours

source	sum of squares SS	degrees of freedom ν ν	mean square MS	F statistic	p-value
treatment	0.1458	7	0.0208	25.3356	1.61E-07
error	0.0132	16	0.0008		
total	0.159	23			

Tukey HSD results

treatments pair	Tukey HSD Q statistic	Tukey HSD p-value	Tukey HSD inferfence
A vs B	14.597	0.001005	** p<0.01
A vs C	14.4158	0.001005	** p<0.01
A vs D	14.1541	0.001005	** p<0.01

6 hours

source	sum of squares SS	degrees of freedom ν ν	mean square MS	F statistic	p-value
treatment	0.1432	7	0.0205	22.6315	3.60E-07
error	0.0145	16	0.0009		
total	0.1576	23			

Tukey HSD results

treatments pair	Tukey HSD Q statistic	Tukey HSD p-value	Tukey HSD inferfence
A vs B	13.9627	0.001005	** p<0.01
A vs C	13.6938	0.001005	** p<0.01
A vs D	13.4057	0.001005	** p<0.01

A vs E	14.4158	0.001005	** p<0.01
A vs F	14.4158	0.001005	** p<0.01
A vs G	14.1541	0.001005	** p<0.01
A vs H	13.0467	0.001005	** p<0.01
B vs C	0.1812	0.899995	insignificant
B vs D	0.4429	0.899995	insignificant
B vs E	0.1812	0.899995	insignificant
B vs F	0.1812	0.899995	insignificant
B vs G	0.4429	0.899995	insignificant
B vs H	1.5503	0.899995	insignificant
C vs D	0.2617	0.899995	insignificant
C vs E	0	0.899995	insignificant
C vs F	0	0.899995	insignificant
C vs G	0.2617	0.899995	insignificant
C vs H	1.3691	0.899995	insignificant
D vs E	0.2617	0.899995	insignificant
D vs F	0.2617	0.899995	insignificant
D vs G	0	0.899995	insignificant
D vs H	1.1074	0.899995	insignificant
E vs F	0	0.899995	insignificant
E vs G	0.2617	0.899995	insignificant
E vs H	1.3691	0.899995	insignificant
F vs G	0.2617	0.899995	insignificant
F vs H	1.3691	0.899995	insignificant
G vs H	1.1074	0.899995	insignificant

12 hours

A vs E	13.7514	0.001005	** p<0.01
A vs F	13.7514	0.001005	** p<0.01
A vs G	12.4454	0.001005	** p<0.01
A vs H	12.4454	0.001005	** p<0.01
B vs C	0.2689	0.899995	insignificant
B vs D	0.557	0.899995	insignificant
B vs E	0.2113	0.899995	insignificant
B vs F	0.2113	0.899995	insignificant
B vs G	1.5173	0.899995	insignificant
B vs H	1.5173	0.899995	insignificant
C vs D	0.2881	0.899995	insignificant
C vs E	0.0576	0.899995	insignificant
C vs F	0.0576	0.899995	insignificant
C vs G	1.2484	0.899995	insignificant
C vs H	1.2484	0.899995	insignificant
D vs E	0.3457	0.899995	insignificant
D vs F	0.3457	0.899995	insignificant
D vs G	0.9603	0.899995	insignificant
D vs H	0.9603	0.899995	insignificant
E vs F	0	0.899995	insignificant
E vs G	1.306	0.899995	insignificant
E vs H	1.306	0.899995	insignificant
F vs G	1.306	0.899995	insignificant
F vs H	1.306	0.899995	insignificant
G vs H	0	0.899995	insignificant

12 hours

source	sum of squares SS	degrees of freedom ν ν	mean square MS	F statistic	p-value
treatment	1.0755	7	0.1536	223.7156	9.55E-15
error	0.011	16	0.0007		
total	1.0864	23			

source	sum of squares SS	degrees of freedom ν ν	mean square MS	F statistic	p-value
treatment	1.0976	7	0.1568	518.9928	1.11E-16
error	0.0048	16	0.0003		
total	1.1024	23			

Tukey HSD results

treatments pair	Tukey HSD Q statistic	Tukey HSD p-value	Tukey HSD inferfence
A vs B	44.6574	0.001005	** p<0.01
A vs C	44.4812	0.001005	** p<0.01
A vs D	42.1238	0.001005	** p<0.01
A vs E	43.9965	0.001005	** p<0.01
A vs F	40.4054	0.001005	** p<0.01
A vs G	37.5413	0.001005	** p<0.01
A vs H	35.8229	0.001005	** p<0.01
B vs C	0.1763	0.899995	insignificant
B vs D	2.5336	0.619908	insignificant
B vs E	0.6609	0.899995	insignificant
B vs F	4.252	0.114568	insignificant
B vs G	7.1161	0.002406	** p<0.01
B vs H	8.8345	0.001005	** p<0.01
C vs D	2.3573	0.685871	insignificant
C vs E	0.4847	0.899995	insignificant
C vs F	4.0758	0.141707	insignificant
C vs G	6.9399	0.003062	** p<0.01
C vs H	8.6583	0.001005	** p<0.01
D vs E	1.8727	0.867277	insignificant
D vs F	1.7184	0.899995	insignificant

Tukey HSD results

treatments pair	Tukey HSD Q statistic	Tukey HSD p-value	Tukey HSD inferfence
A vs B	67.3286	0.001005	** p<0.01
A vs C	66.93	0.001005	** p<0.01
A vs D	66.4318	0.001005	** p<0.01
A vs E	60.9512	0.001005	** p<0.01
A vs F	66.3322	0.001005	** p<0.01
A vs G	54.0091	0.001005	** p<0.01
A vs H	49.8571	0.001005	** p<0.01
B vs C	0.3986	0.899995	insignificant
B vs D	0.8968	0.899995	insignificant
B vs E	6.3775	0.006632	** p<0.01
B vs F	0.9965	0.899995	insignificant
B vs G	13.3196	0.001005	** p<0.01
B vs H	17.4716	0.001005	** p<0.01
C vs D	0.4982	0.899995	insignificant
C vs E	5.9789	0.011487	* p<0.05
C vs F	0.5979	0.899995	insignificant
C vs G	12.921	0.001005	** p<0.01
C vs H	17.073	0.001005	** p<0.01
D vs E	5.4806	0.022768	* p<0.05
D vs F	0.0996	0.899995	insignificant

D vs G	4.5825	0.075504	insignificant
D vs H	6.301	0.007369	** p<0.01
E vs F	3.5911	0.247093	insignificant
E vs G	6.4552	0.005957	** p<0.01
E vs H	8.1736	0.001005	** p<0.01
F vs G	2.8641	0.496225	insignificant
F vs H	4.5825	0.075504	insignificant
G vs H	1.7184	0.899995	insignificant

D vs G	12.4227	0.001005	** p<0.01
D vs H	16.5747	0.001005	** p<0.01
E vs F	5.381	0.026081	* p<0.05
E vs G	6.9421	0.003055	** p<0.01
E vs H	11.0941	0.001005	** p<0.01
F vs G	12.3231	0.001005	** p<0.01
F vs H	16.4751	0.001005	** p<0.01
G vs H	4.152	0.12911	insignificant

18 hours

source	sum of squares SS	degrees of freedom ν	mean square MS	F statistic	p-value
treatment	2.4642	7	0.352	100.8511	4.92E-12
error	0.0559	16	0.0035		
total	2.5201	23			

Tukey HSD results

treatments pair	Tukey HSD Q statistic	Tukey HSD p-value	Tukey HSD inferfence
A vs B	29.2088	0.001005	** p<0.01
A vs C	29.1306	0.001005	** p<0.01
A vs D	28.085	0.001005	** p<0.01
A vs E	27.958	0.001005	** p<0.01
A vs F	24.1371	0.001005	** p<0.01
A vs G	16.1533	0.001005	** p<0.01
A vs H	19.0849	0.001005	** p<0.01
B vs C	0.0782	0.899995	insignificant
B vs D	1.1238	0.899995	insignificant

18 hours

source	sum of squares SS	degrees of freedom ν	mean square MS	F statistic	p-value
treatment	2.5412	7	0.363	171.5092	7.72E-14
error	0.0339	16	0.0021		
total	2.5751	23			

Tukey HSD results

treatments pair	Tukey HSD Q statistic	Tukey HSD p-value	Tukey HSD inferfence
A vs B	37.5093	0.001005	** p<0.01
A vs C	37.3587	0.001005	** p<0.01
A vs D	37.1705	0.001005	** p<0.01
A vs E	34.7736	0.001005	** p<0.01
A vs F	36.0285	0.001005	** p<0.01
A vs G	23.2535	0.001005	** p<0.01
A vs H	23.0151	0.001005	** p<0.01
B vs C	0.1506	0.899995	insignificant
B vs D	0.3388	0.899995	insignificant

B vs E	1.2508	0.899995	insignificant
B vs F	5.0717	0.039611	* p<0.05
B vs G	13.0555	0.001005	** p<0.01
B vs H	10.1239	0.001005	** p<0.01
C vs D	1.0456	0.899995	insignificant
C vs E	1.1727	0.899995	insignificant
C vs F	4.9935	0.043968	* p<0.05
C vs G	12.9773	0.001005	** p<0.01
C vs H	10.0457	0.001005	** p<0.01
D vs E	0.127	0.899995	insignificant
D vs F	3.9479	0.165241	insignificant
D vs G	11.9317	0.001005	** p<0.01
D vs H	9.0001	0.001005	** p<0.01
E vs F	3.8209	0.191251	insignificant
E vs G	11.8047	0.001005	** p<0.01
E vs H	8.8731	0.001005	** p<0.01
F vs G	7.9838	0.001005	** p<0.01
F vs H	5.0522	0.040658	* p<0.05
G vs H	2.9316	0.470692	insignificant

24 hours

source	sum of squares SS	degrees of freedom ν	mean square MS	F statistic	p-value
treatment	4.9242	7	0.7035	375.9904	1.11E-16
error	0.0299	16	0.0019		
total	4.9542	23			

Tukey HSD results

treatments	Tukey HSD	Tukey HSD	Tukey HSD

B vs E	2.7357	0.544263	insignificant
B vs F	1.4808	0.899995	insignificant
B vs G	14.2558	0.001005	** p<0.01
B vs H	14.4942	0.001005	** p<0.01
C vs D	0.1882	0.899995	insignificant
C vs E	2.5851	0.600626	insignificant
C vs F	1.3302	0.899995	insignificant
C vs G	14.1052	0.001005	** p<0.01
C vs H	14.3436	0.001005	** p<0.01
D vs E	2.3969	0.671077	insignificant
D vs F	1.142	0.899995	insignificant
D vs G	13.917	0.001005	** p<0.01
D vs H	14.1554	0.001005	** p<0.01
E vs F	1.2549	0.899995	insignificant
E vs G	11.5201	0.001005	** p<0.01
E vs H	11.7585	0.001005	** p<0.01
F vs G	12.775	0.001005	** p<0.01
F vs H	13.0134	0.001005	** p<0.01
G vs H	0.2384	0.899995	insignificant

24 hours

source	sum of squares SS	degrees of freedom ν	mean square MS	F statistic	p-value
treatment	6.1131	7	0.8733	559.4764	1.11E-16
error	0.025	16	0.0016		
total	6.138	23			

Tukey HSD results

treatments	Tukey HSD	Tukey HSD	Tukey HSD

pair	Q statistic	p-value	inferfence
A vs B	45.7694	0.001005	** p<0.01
A vs C	45.7026	0.001005	** p<0.01
A vs D	44.2744	0.001005	** p<0.01
A vs E	42.0987	0.001005	** p<0.01
A vs F	33.3026	0.001005	** p<0.01
A vs G	8.5826	0.001005	** p<0.01
A vs H	9.2099	0.001005	** p<0.01
B vs C	0.0667	0.899995	insignificant
B vs D	1.4949	0.899995	insignificant
B vs E	3.6706	0.22665	insignificant
B vs F	12.4668	0.001005	** p<0.01
B vs G	37.1868	0.001005	** p<0.01
B vs H	36.5594	0.001005	** p<0.01
C vs D	1.4282	0.899995	insignificant
C vs E	3.6039	0.243672	insignificant
C vs F	12.4	0.001005	** p<0.01
C vs G	37.12	0.001005	** p<0.01
C vs H	36.4927	0.001005	** p<0.01
D vs E	2.1757	0.753867	insignificant
D vs F	10.9718	0.001005	** p<0.01
D vs G	35.6918	0.001005	** p<0.01
D vs H	35.0645	0.001005	** p<0.01
E vs F	8.7962	0.001005	** p<0.01
E vs G	33.5161	0.001005	** p<0.01
E vs H	32.8888	0.001005	** p<0.01
F vs G	24.72	0.001005	** p<0.01
F vs H	24.0926	0.001005	** p<0.01
G vs H	0.6273	0.899995	insignificant

pair	Q statistic	p-value	inferfence
A vs B	52.9587	0.001005	** p<0.01
A vs C	52.8857	0.001005	** p<0.01
A vs D	51.3221	0.001005	** p<0.01
A vs E	46.2512	0.001005	** p<0.01
A vs F	33.5084	0.001005	** p<0.01
A vs G	6.1814	0.008688	** p<0.01
A vs H	4.0479	0.146596	insignificant
B vs C	0.0731	0.899995	insignificant
B vs D	1.6367	0.899995	insignificant
B vs E	6.7075	0.004209	** p<0.01
B vs F	19.4504	0.001005	** p<0.01
B vs G	46.7773	0.001005	** p<0.01
B vs H	48.9109	0.001005	** p<0.01
C vs D	1.5636	0.899995	insignificant
C vs E	6.6345	0.004657	** p<0.01
C vs F	19.3773	0.001005	** p<0.01
C vs G	46.7042	0.001005	** p<0.01
C vs H	48.8378	0.001005	** p<0.01
D vs E	5.0708	0.039655	* p<0.05
D vs F	17.8137	0.001005	** p<0.01
D vs G	45.1406	0.001005	** p<0.01
D vs H	47.2742	0.001005	** p<0.01
E vs F	12.7428	0.001005	** p<0.01
E vs G	40.0698	0.001005	** p<0.01
E vs H	42.2033	0.001005	** p<0.01
F vs G	27.3269	0.001005	** p<0.01
F vs H	29.4605	0.001005	** p<0.01
G vs H	2.1335	0.769636	insignificant

30 hours

source	sum of squares SS	degrees of freedom ν ν	mean square MS	F statistic	p-value
treatment	6.1131	7	0.8733	559.4764	1.11E-16
error	0.025	16	0.0016		
total	6.138	23			

Tukey HSD results

treatments pair	Tukey HSD Q statistic	Tukey HSD p-value	Tukey HSD inferfence
A vs B	52.9587	0.001005	** p<0.01
A vs C	52.8857	0.001005	** p<0.01
A vs D	51.3221	0.001005	** p<0.01
A vs E	46.2512	0.001005	** p<0.01
A vs F	33.5084	0.001005	** p<0.01
A vs G	6.1814	0.008688	** p<0.01
A vs H	4.0479	0.146596	insignificant
B vs C	0.0731	0.899995	insignificant
B vs D	1.6367	0.899995	insignificant
B vs E	6.7075	0.004209	** p<0.01
B vs F	19.4504	0.001005	** p<0.01
B vs G	46.7773	0.001005	** p<0.01
B vs H	48.9109	0.001005	** p<0.01
C vs D	1.5636	0.899995	insignificant
C vs E	6.6345	0.004657	** p<0.01
C vs F	19.3773	0.001005	** p<0.01
C vs G	46.7042	0.001005	** p<0.01
C vs H	48.8378	0.001005	** p<0.01

30 hours

source	sum of squares SS	degrees of freedom ν ν	mean square MS	F statistic	p-value
treatment	5.1763	7	0.7395	269.2062	2.22E-15
error	0.0439	16	0.0027		
total	5.2203	23			

Tukey HSD results

treatments pair	Tukey HSD Q statistic	Tukey HSD p-value	Tukey HSD inferfence
A vs B	39.8886	0.001005	** p<0.01
A vs C	39.8225	0.001005	** p<0.01
A vs D	39.6572	0.001005	** p<0.01
A vs E	36.5067	0.001005	** p<0.01
A vs F	32.2766	0.001005	** p<0.01
A vs G	13.1199	0.001005	** p<0.01
A vs H	8.0416	0.001005	** p<0.01
B vs C	0.0661	0.899995	insignificant
B vs D	0.2313	0.899995	insignificant
B vs E	3.3819	0.308022	insignificant
B vs F	7.612	0.001231	** p<0.01
B vs G	26.7686	0.001005	** p<0.01
B vs H	31.847	0.001005	** p<0.01
C vs D	0.1652	0.899995	insignificant
C vs E	3.3158	0.329071	insignificant
C vs F	7.5459	0.001344	** p<0.01
C vs G	26.7025	0.001005	** p<0.01
C vs H	31.7809	0.001005	** p<0.01

D vs E	5.0708	0.039655	* p<0.05
D vs F	17.8137	0.001005	** p<0.01
D vs G	45.1406	0.001005	** p<0.01
D vs H	47.2742	0.001005	** p<0.01
E vs F	12.7428	0.001005	** p<0.01
E vs G	40.0698	0.001005	** p<0.01
E vs H	42.2033	0.001005	** p<0.01
F vs G	27.3269	0.001005	** p<0.01
F vs H	29.4605	0.001005	** p<0.01
G vs H	2.1335	0.769636	insignificant

48 hours

source	sum of squares SS	degrees of freedom ν *ν*	mean square MS	F statistic	p-value
treatment	6.5488	7	0.9355	397.2665	1.11E-16
error	0.0377	16	0.0024		
total	6.5865	23			

Tukey HSD results

treatments pair	Tukey HSD Q statistic	Tukey HSD p-value	Tukey HSD inferfence
A vs B	44.008	0.001005	** p<0.01
A vs C	43.9843	0.001005	** p<0.01
A vs D	42.7112	0.001005	** p<0.01
A vs E	37.9523	0.001005	** p<0.01
A vs F	25.1746	0.001005	** p<0.01
A vs G	3.9618	0.162549	insignificant
A vs H	2.4151	0.664239	insignificant

D vs E	3.1505	0.387605	insignificant
D vs F	7.3807	0.001679	** p<0.01
D vs G	26.5373	0.001005	** p<0.01
D vs H	31.6156	0.001005	** p<0.01
E vs F	4.2301	0.117738	insignificant
E vs G	23.3868	0.001005	** p<0.01
E vs H	28.4651	0.001005	** p<0.01
F vs G	19.1566	0.001005	** p<0.01
F vs H	24.235	0.001005	** p<0.01
G vs H	5.0783	0.039259	* p<0.05

48 hours

source	sum of squares SS	degrees of freedom ν *ν*	mean square MS	F statistic	p-value
treatment	6.5488	7	0.9355	397.2665	1.11E-16
error	0.0377	16	0.0024		
total	6.5865	23			

Tukey HSD results

treatments pair	Tukey HSD Q statistic	Tukey HSD p-value	Tukey HSD inferfence
A vs B	44.008	0.001005	** p<0.01
A vs C	43.9843	0.001005	** p<0.01
A vs D	42.7112	0.001005	** p<0.01
A vs E	37.9523	0.001005	** p<0.01
A vs F	25.1746	0.001005	** p<0.01
A vs G	3.9618	0.162549	insignificant
A vs H	2.4151	0.664239	insignificant

B vs C	0.0238	0.899995	insignificant
B vs D	1.2968	0.899995	insignificant
B vs E	6.0557	0.010334	* p<0.05
B vs F	18.8334	0.001005	** p<0.01
B vs G	40.0463	0.001005	** p<0.01
B vs H	41.5929	0.001005	** p<0.01
C vs D	1.273	0.899995	insignificant
C vs E	6.0319	0.01068	* p<0.05
C vs F	18.8096	0.001005	** p<0.01
C vs G	40.0225	0.001005	** p<0.01
C vs H	41.5691	0.001005	** p<0.01
D vs E	4.7589	0.059986	insignificant
D vs F	17.5366	0.001005	** p<0.01
D vs G	38.7494	0.001005	** p<0.01
D vs H	40.2961	0.001005	** p<0.01
E vs F	12.7777	0.001005	** p<0.01
E vs G	33.9905	0.001005	** p<0.01
E vs H	35.5372	0.001005	** p<0.01
F vs G	21.2129	0.001005	** p<0.01
F vs H	22.7595	0.001005	** p<0.01
G vs H	1.5466	0.899995	insignificant

B vs C	0.0238	0.899995	insignificant
B vs D	1.2968	0.899995	insignificant
B vs E	6.0557	0.010334	* p<0.05
B vs F	18.8334	0.001005	** p<0.01
B vs G	40.0463	0.001005	** p<0.01
B vs H	41.5929	0.001005	** p<0.01
C vs D	1.273	0.899995	insignificant
C vs E	6.0319	0.01068	* p<0.05
C vs F	18.8096	0.001005	** p<0.01
C vs G	40.0225	0.001005	** p<0.01
C vs H	41.5691	0.001005	** p<0.01
D vs E	4.7589	0.059986	insignificant
D vs F	17.5366	0.001005	** p<0.01
D vs G	38.7494	0.001005	** p<0.01
D vs H	40.2961	0.001005	** p<0.01
E vs F	12.7777	0.001005	** p<0.01
E vs G	33.9905	0.001005	** p<0.01
E vs H	35.5372	0.001005	** p<0.01
F vs G	21.2129	0.001005	** p<0.01
F vs H	22.7595	0.001005	** p<0.01
G vs H	1.5466	0.899995	insignificant

Table S3. Resistance of tested LAB strains in simulated *in vitro* gastric (t < 2 h) and intestinal (t > 2 h) conditions.

ORE **L. acidophillus+sugar syrup** ***L. acidophillus*** **glucose+fructose syrup**

source	sum of squares SS	degrees of freedom νν	mean square MS	F statistic	p-value
treatment	63.5042	5	12.7008	17.144	0.0017
error	4.445	6	0.7408		
total	67.9492	11			

Tukey HSD results

treatments pair	Tukey HSD Q statistic	Tukey HSD p-value	Tukey HSD inferfence
A vs B	2.4646	0.552622	insignificant
A vs C	6.5723	0.02528	* p<0.05
A vs D	0	0.899995	insignificant
A vs E	3.5326	0.256811	insignificant
A vs F	10.6799	0.002189	** p<0.01
B vs C	4.1077	0.163306	insignificant
B vs D	2.4646	0.552622	insignificant
B vs E	1.068	0.899995	insignificant
B vs F	8.2153	0.008613	** p<0.01
C vs D	6.5723	0.02528	* p<0.05
C vs E	3.0397	0.374005	insignificant
C vs F	4.1077	0.163306	insignificant
D vs E	3.5326	0.256811	insignificant
D vs F	10.6799	0.002189	** p<0.01
E vs F	7.1473	0.017059	* p<0.05

ORE *L. plantarum*+ sugar syrup *L. plantarum*+ glucose+fructose syrup

source	sum of	degrees of	mean square	F statistic	p-value

	squares SS	freedom *vv*	MS		
treatment	61.311	5	12.2622	60.2442	4.78E-05
error	1.2212	6	0.2035		
total	62.5323	11			

Tukey HSD results

treatments pair	Tukey HSD Q statistic	Tukey HSD p-value	Tukey HSD inferfence
A vs B	2.351	0.590576	insignificant
A vs C	10.0309	0.003063	** p<0.01
A vs D	0	0.899995	insignificant
A vs E	12.4602	0.001005	** p<0.01
A vs F	18.9646	0.001005	** p<0.01
B vs C	7.6799	0.012044	* p<0.05
B vs D	2.351	0.590576	insignificant
B vs E	10.1092	0.002939	** p<0.01
B vs F	16.6136	0.001005	** p<0.01
C vs D	10.0309	0.003063	** p<0.01
C vs E	2.4293	0.564397	insignificant
C vs F	8.9337	0.005618	** p<0.01
D vs E	12.4602	0.001005	** p<0.01
D vs F	18.9646	0.001005	** p<0.01
E vs F	6.5044	0.026512	* p<0.05

ORE *A. kunkeei*+ sugar syrup *A. kunkeei*+ glucose+fructose syrup

source	sum of squares SS	degrees of freedom *vv*	mean square MS	F statistic	p-value
treatment	28.4942	5	5.6988	16.6389	0.0018
error	2.055	6	0.3425		
total	30.5492	11			

Tukey HSD results

treatments pair	Tukey HSD Q statistic	Tukey HSD p-value	Tukey HSD inferfence
A vs B	1.8124	0.770496	insignificant
A vs C	5.6787	0.048214	* $p<0.05$
A vs D	0	0.899995	insignificant
A vs E	6.4037	0.028459	* $p<0.05$
A vs F	10.1493	0.002878	** $p<0.01$
B vs C	3.8664	0.197621	insignificant
B vs D	1.8124	0.770496	insignificant
B vs E	4.5913	0.11134	insignificant
B vs F	8.3369	0.007998	** $p<0.01$
C vs D	5.6787	0.048214	* $p<0.05$
C vs E	0.7249	0.899995	insignificant
C vs F	4.4705	0.122508	insignificant
D vs E	6.4037	0.028459	* $p<0.05$
D vs F	10.1493	0.002878	** $p<0.01$
E vs F	3.7456	0.217423	insignificant

ORE *LAB mix* + sugar syrup *LAB mix* + glucose+fructose syrup

source	sum of squares SS	degrees of freedom νν	mean square MS	F statistic	p-value
treatment	20.006	5	4.0012	30.7608	0.0003
error	0.7804	6	0.1301		
total	20.7865	11			

Tukey HSD results

treatments pair	Tukey HSD Q statistic	Tukey HSD p-value	Tukey HSD inferfence
A vs B	2.9409	0.402263	insignificant
A vs C	8.7639	0.006201	** $p<0.01$

A vs D	0	0.899995	insignificant
A vs E	8.4306	0.00756	** p<0.01
A vs F	13.7242	0.001005	** p<0.01
B vs C	5.823	0.043312	* p<0.05
B vs D	2.9409	0.402263	insignificant
B vs E	5.4897	0.055564	insignificant
B vs F	10.7833	0.002078	** p<0.01
C vs D	8.7639	0.006201	** p<0.01
C vs E	0.3333	0.899995	insignificant
C vs F	4.9603	0.08337	insignificant
D vs E	8.4306	0.00756	** p<0.01
D vs F	13.7242	0.001005	** p<0.01
E vs F	5.2936	0.064481	insignificant

Table S4. Comparison of capacities of biofilm formation by tested LAB. Influence of MRS medium supplementation with sucrose, glucose, and fructose syrups on biofilm formation.

L. plantarum

source	sum of squares SS	degrees of freedom $\nu\nu$	mean square MS	F statistic	p-value
treatment	0.0512	2	0.0256	4.9839	0.0531
error	0.0308	6	0.0051		
total	0.082	8			

Tukey HSD results

treatments pair	Tukey HSD Q statistic	Tukey HSD p-value	Tukey HSD inferfence
A vs B	1.4182	0.599839	insignificant
A vs C	2.9574	0.17154	insignificant
B vs C	4.3756	0.048221	* p<0.05

L. acidophillus

source	sum of squares SS	degrees of freedom νν	mean square MS	F statistic	p-value
treatment	0.0296	2	0.0148	2.3293	0.1784
error	0.0381	6	0.0063		
total	0.0676	8			

Tukey HSD results

treatments pair	Tukey HSD Q statistic	Tukey HSD p-value	Tukey HSD inferfence
A vs B	1.3338	0.631292	insignificant
A vs C	1.7108	0.49118	insignificant
B vs C	3.0446	0.158557	insignificant

L. kunkeei

source	sum of squares SS	degrees of freedom νν	mean square MS	F statistic	p-value
treatment	0.0686	2	0.0343	8.4159	0.0181
error	0.0245	6	0.0041		
total	0.0931	8			

Tukey HSD results

treatments pair	Tukey HSD Q statistic	Tukey HSD p-value	Tukey HSD inferfence
A vs B	2.6581	0.224061	insignificant
A vs C	3.1373	0.145801	insignificant
B vs C	5.7954	0.014954	* p<0.05

LAB mix

source	sum of squares SS	degrees of freedom $\nu\nu$	mean square MS	F statistic	p-value
treatment	0.0674	2	0.0337	5.3221	0.0468
error	0.038	6	0.0063		
total	0.1054	8			

Tukey HSD results

treatments pair	Tukey HSD Q statistic	Tukey HSD p-value	Tukey HSD inferfence
A vs B	1.2406	0.666051	insignificant
A vs C	3.2284	0.134254	insignificant
B vs C	4.4689	0.044466	* p<0.05

MRS

One-way ANOVA of your kk=4 independent treatments:

source	sum of squares SS	degrees of freedom $\nu\nu$	mean square MS	F statistic	p-value
treatment	0.1206	3	0.0402	22.2398	0.0003
error	0.0145	8	0.0018		
total	0.135	11			

Tukey HSD results

treatments pair	Tukey HSD Q statistic	Tukey HSD p-value	Tukey HSD inferfence
A vs B	5.1877	0.026147	* p<0.05
A vs C	3.0692	0.211136	insignificant

A vs D	5.663	0.016562	* p<0.05
B vs C	2.1185	0.481706	insignificant
B vs D	10.8507	0.001005	** p<0.01
C vs D	8.7322	0.001205	** p<0.01

MRS+sugar syrup

source	sum of squares SS	degrees of freedom νν	mean square MS	F statistic	p-value
treatment	0.1414	3	0.047	5.628	0.023
error	0.067	8	0.008		
total	0.2084	11			

treatments pair	Tukey HSD Q statistic	Tukey HSD p-value	Tukey HSD inferfence
A vs B	2.4604	0.366	insignificant
A vs C	2.1702	0.463	insignificant
A vs D	2.6623	0.307	insignificant
B vs C	0.2902	0.9	insignificant
B vs D	5.1227	0.028	* p<0.05
C vs D	4.8325	0.037	* p<0.05

MRS+glucose+fructose syrup

source	sum of squares SS	degrees of freedom νν	mean square MS	F statistic	p-value
treatment	0.1838	3	0.061	9.827	0.005
error	0.0499	8	0.006		
total	0.2336	11			

Tukey HSD results

treatments pair	Tukey HSD Q statistic	Tukey HSD p-value	Tukey HSD inferfence
A vs B	3.7514	0.109	insignificant
A vs C	1.7989	0.597	insignificant
A vs D	3.6198	0.124	insignificant
B vs C	1.9525	0.541	insignificant
B vs D	7.3712	0.004	** p<0.01
C vs D	5.4187	0.021	* p<0.05

Table S5 . Growth inhibition (%) of tested patogenic strains by LAB supernatants at physiological pH of LAB evaluated by microplate method.

C. albicans 50%

source	sum of squares SS	degrees of freedom νν	mean square MS	F statistic	p-value
treatment	718.482	3	239.494	236.5687	5.88E-05
error	4.0495	4	1.0124		
total	######	7			

treatments pair	Tukey HSD Q statistic	Tukey HSD p-value	Tukey HSD inferfence
A vs B	19.6988	0.0010053	** p<0.01
A vs C	9.8986	0.0075655	** p<0.01
A vs D	36.0875	0.0010053	** p<0.01
B vs C	9.8002	0.0078508	** p<0.01
B vs D	16.3887	0.0011112	** p<0.01
C vs D	26.1889	0.0010053	** p<0.01

C. albicans 25%

source	sum of squares SS	degrees of freedom νν	mean square MS	F statistic	p-value
treatment	442.298	3	147.4327	79.7791	0.0005
error	7.392	4	1.848		
total	449.69	7			

treatments pair	Tukey HSD Q statistic	Tukey HSD p-value	Tukey HSD inferfence
A vs B	17.6489	0.0010053	** $p<0.01$
A vs C	11.0023	0.0051044	** $p<0.01$
A vs D	19.9063	0.0010053	** $p<0.01$
B vs C	6.6465	0.03115	* $p<0.05$
B vs D	2.2575	0.4715737	insignificant
C vs D	8.904	0.0111525	* $p<0.05$

C.albicans *12.5%*

source	sum of squares SS	degrees of freedom νν	mean square MS	F statistic	p-value
treatment	83.8145	3	27.9382	17.0956	0.0096
error	6.5369	4	1.6342		
total	90.3515	7			

treatments pair	Tukey HSD Q statistic	Tukey HSD p-value	Tukey HSD inferfence
A vs B	3.3293	0.2288209	insignificant
A vs C	2.2551	0.4722758	insignificant

A vs D	5.9285	0.045502	* p<0.05
B vs C	1.0742	0.8599734	insignificant
B vs D	9.2577	0.009679	** p<0.01
C vs D	8.1836	0.015109	* p<0.05

B. cereus 50%

source	sum of squares SS	degrees of freedom $\nu\nu$	mean square MS	F statistic	p-value
treatment	204.6466	3	68.2155	31.3743	0.0031
error	8.697	4	2.1742		
total	213.3436	7			

treatments pair	Tukey HSD Q statistic	Tukey HSD p-value	Tukey HSD inferfence
A vs B	4.9585	0.0795427	insignificant
A vs C	1.5633	0.6982316	insignificant
A vs D	8.5935	0.0126776	* p<0.05
B vs C	6.5218	0.0332001	* p<0.05
B vs D	13.552	0.0023162	** p<0.01
C vs D	7.0301	0.0257325	* p<0.05

B. cereus 25%

source	sum of squares SS	degrees of freedom $\nu\nu$	mean square MS	F statistic	p-value
treatment	369.1873	3	123.0624	65.6714	0.0007
error	7.4956	4	1.8739		
total	376.683	7			

treatments pair	Tukey HSD Q statistic	Tukey HSD p-value	Tukey HSD inferfence
A vs B	16.5554	0.0010688	** p<0.01

A vs C	1.8182	0.6139413	insignificant
A vs D	0.4236	0.8999947	insignificant
B vs C	14.7371	0.0016786	** p<0.01
B vs D	16.9789	0.0010053	** p<0.01
C vs D	2.2418	0.476231	insignificant

B. cereus *12.5%*

source	sum of squares SS	degrees of freedom $\nu\nu$	mean square MS	F statistic	p-value
treatment	4.4427	3	1.4809	4.4703	0.091
error	1.3251	4	0.3313		
total	5.7678	7			

Ps. aerug. 50%

source	sum of squares SS	degrees of freedom $\nu\nu$	mean square MS	F statistic	p-value
treatment	776.4296	3	258.8099	80.0361	0.0005
error	12.9346	4	3.2337		
total	789.3643	7			

treatments pair	Tukey HSD Q statistic	Tukey HSD p-value	Tukey HSD inferfence
A vs B	7.8133	0.017803	* p<0.05
A vs C	9.2486	0.0097149	** p<0.01
A vs D	21.6272	0.0010053	** p<0.01
B vs C	1.4353	0.7405772	insignificant
B vs D	13.8139	0.0021515	** p<0.01
C vs D	12.3786	0.0032731	** p<0.01

Ps. aerug. 25%

source	sum of squares SS	degrees of freedom $\nu\nu$	mean square MS	F statistic	p-value
treatment	180.3105	3	60.1035	33.4791	0.0027
error	7.181	4	1.7953		
total	187.4915	7			

treatments pair	Tukey HSD Q statistic	Tukey HSD p-value	Tukey HSD inferfence
A vs B	4.3486	0.1163285	insignificant
A vs C	0.5726	0.8999947	insignificant
A vs D	9.4466	0.0089873	** p<0.01
B vs C	4.9212	0.0813633	insignificant
B vs D	13.7952	0.0021655	** p<0.01
C vs D	8.874	0.0112913	* p<0.05

Ps. aerug. 12.5%

source	sum of squares SS	degrees of freedom $\nu\nu$	mean square MS	F statistic	p-value
treatment	0.7755	3	0.2585	5.3819	0.0688
error	0.1921	4	0.048		
total	0.9676	7			

S.aureus 50%

source	sum of squares SS	degrees of freedom $\nu\nu$	mean square MS	F statistic	p-value
treatment	366.5784	3	122.1928	33.4438	0.0027
error	14.6147	4	3.6537		
total	381.1932	7			

treatments pair	Tukey HSD Q statistic	Tukey HSD p-value	Tukey HSD inferfence
A vs B	1.9717	0.5631915	insignificant
A vs C	1.6092	0.6830644	insignificant
A vs D	11.0683	0.0049934	** p<0.01
B vs C	3.5809	0.1928799	insignificant
B vs D	13.04	0.0026847	** p<0.01
C vs D	9.4591	0.0089436	** p<0.01

S.aureus 25%

source	sum of squares SS	degrees of freedom $\nu\nu$	mean square MS	F statistic	p-value
treatment	209.5826	3	69.8609	18.1738	0.0086
error	15.3762	4	3.844		
total	224.9587	7			

treatments pair	Tukey HSD Q statistic	Tukey HSD p-value	Tukey HSD inferfence
A vs B	2.4885	0.4057703	insignificant
A vs C	2.0052	0.5521094	insignificant
A vs D	6.7514	0.0295411	* p<0.05
B vs C	0.4833	0.8999947	insignificant
B vs D	9.2399	0.0097487	** p<0.01
C vs D	8.7567	0.011846	* p<0.05

S.aureus 12.5%

source	sum of squares SS	degrees of freedom $\nu\nu$	mean square MS	F statistic	p-value
treatment	7.2007	3	2.4002	25.4214	0.0046
error	0.3777	4	0.0944		
total	7.5783	7			

treatments pair	Tukey HSD Q statistic	Tukey HSD p-value	Tukey HSD inferfence
A vs B	6.0729	0.0420586	* p<0.05
A vs C	0.5638	0.8999947	insignificant
A vs D	10.6777	0.00571	** p<0.01
B vs C	5.5091	0.0575456	insignificant
B vs D	4.6048	0.0989274	insignificant
C vs D	10.1139	0.0069881	** p<0.01

S. enteritidis
50%

source	sum of squares SS	degrees of freedom $\nu\nu$	mean square MS	F statistic	p-value
treatment	100.5676	3	33.5225	10.2837	0.0237
error	13.0391	4	3.2598		
total	113.6067	7			

treatments pair	Tukey HSD Q statistic	Tukey HSD p-value	Tukey HSD inferfence
A vs B	4.2611	0.123057	insignificant
A vs C	5.064	0.0746536	insignificant
A vs D	1.5157	0.7139927	insignificant
B vs C	0.8029	0.8999947	insignificant
B vs D	5.7767	0.0494914	* p<0.05
C vs D	6.5796	0.0322298	* p<0.05

S. enteritidis
25%

source	sum of squares SS	degrees of freedom $\nu\nu$	mean square MS	F statistic	p-value
treatment	32.4312	3	10.8104	6.8932	0.0465
error	6.2731	4	1.5683		

total	38.7043	7			

treatments pair	Tukey HSD Q statistic	Tukey HSD p-value	Tukey HSD inferfence
A vs B	2.5065	0.4009312	insignificant
A vs C	0.786	0.8999947	insignificant
A vs D	3.7182	0.1758568	insignificant
B vs C	1.7205	0.6462697	insignificant
B vs D	6.2246	0.0387769	* p<0.05
C vs D	4.5042	0.1053806	insignificant

S. enteritidis
12.5%

source	sum of squares SS	degrees of freedom $\nu\nu$	mean square MS	F statistic	p-value
treatment	8.2863	3	2.7621	18.2025	0.0085
error	0.607	4	0.1517		
total	8.8932	7			

treatments pair	Tukey HSD Q statistic	Tukey HSD p-value	Tukey HSD inferfence
A vs B	2.0312	0.5435098	insignificant
A vs C	3.2329	0.244328	insignificant
A vs D	9.8596	0.0076768	** p<0.01
B vs C	1.2017	0.8178123	insignificant
B vs D	7.8284	0.0176844	* p<0.05
C vs D	6.6267	0.0314657	* p<0.05

E.coli 50%

source	sum of squares SS	degrees of freedom $\nu\nu$	mean square MS	F statistic	p-value

treatment	1,243.13	3	414.3764	50.7736	0.0012
error	32.645	4	8.1613		
total	1,275.77	7			
treatments pair	Tukey HSD Q statistic	Tukey HSD p-value	Tukey HSD inferfence		
A vs B	10.7613	0.0055478	** p<0.01		
A vs C	4.962	0.0793759	insignificant		
A vs D	16.4542	0.0010962	** p<0.01		
B vs C	5.7993	0.0488708	* p<0.05		
B vs D	5.6929	0.0518654	insignificant		
C vs D	11.4923	0.0043358	** p<0.01		

E.coli 25%

source	sum of squares SS	degrees of freedom $\nu\nu$	mean square MS	F statistic	p-value
treatment	157.0401	3	52.3467	17.6089	0.0091
error	11.8909	4	2.9727		
total	168.931	7			
treatments pair	Tukey HSD Q statistic	Tukey HSD p-value	Tukey HSD inferfence		
A vs B	5.1572	0.0706229	insignificant		
A vs C	0.47	0.8999947	insignificant		
A vs D	8.229	0.0148121	* p<0.05		
B vs C	5.6272	0.0538184	insignificant		
B vs D	3.0718	0.2728289	insignificant		
C vs D	8.699	0.0121348	* p<0.05		

E. coli 13%

source	sum of squares SS	degrees of freedom $\nu\nu$	mean square MS	F statistic	p-value
treatment	5.2858	3	1.7619	18.5401	0.0082
error	0.3801	4	0.095		
total	5.6659	7			

treatments pair	Tukey HSD Q statistic	Tukey HSD p-value	Tukey HSD inferfence
A vs B	4.9041	0.0822141	insignificant
A vs C	0.1743	0.8999947	insignificant
A vs D	8.7874	0.0116973	* p<0.05
B vs C	5.0784	0.0740128	insignificant
B vs D	3.8833	0.1575566	insignificant
C vs D	8.9617	0.0108943	* p<0.05

E. feclais 50%

source	sum of squares SS	degrees of freedom $\nu\nu$	mean square MS	F statistic	p-value
treatment	#####	3	#####	3.1855	0.146
error	#####	4	5.5489		
total	#####	7			

E. feclais 25%

Treatment →	A	B	C	D
Input Data →	20.689<br>17.64	25.672<br>22.345	19.417<br>17.53	28.497<br>25.84

source	sum of squares SS	degrees of freedom $\nu\nu$	mean square MS	F statistic	p-value
treatment	#####	3	#####	8.7882	0.031
error	#####	4	3.8732		
total	#####	7			

treatments pair	Tukey HSD Q statistic	Tukey HSD p-value	Tukey HSD inferfence
A vs B	3.4808	0.2064102	insignificant
A vs C	0.4965	0.8999947	insignificant
A vs D	5.7516	0.0501899	insignificant
B vs C	3.9774	0.1480466	insignificant
B vs D	2.2707	0.4676484	insignificant
C vs D	6.2481	0.038299	* p<0.05

E. feclais 12.5%

12.5%

source	sum of squares SS	degrees of freedom $\nu\nu$	mean square MS	F statistic	p-value
treatment	#####	3	#####	6.8796	0.047
error	6.7149	4	1.6787		
total	#####	7			

treatments pair	Tukey HSD Q statistic	Tukey HSD p-value	Tukey HSD inferfence
A vs B	0.6265	0.8999947	insignificant
A vs C	2.3451	0.4459221	insignificant
A vs D	5.8472	0.0475885	* p<0.05
B vs C	1.7186	0.6468958	insignificant
B vs D	5.2207	0.068023	insignificant
C vs D	3.5021	0.2034544	insignificant

P. larvae
50%

source	sum of squares SS	degrees of freedom vν	mean square MS	F statistic	p-value
treatment	79.57	3	26.52	5.276	0.071
error	20.11	4	5.027		
total	99.67	7			

P. larvae
25%

ource	sum of squares SS	degrees of freedom vν	mean square MS	F statistic	p-value
treatment	24.3	3	8.101	2.41	0.207
error	13.45	4	3.361		
total	37.75	7			

P. larvae
12.5%

source	sum of squares SS	degrees of freedom vν	mean square MS	F statistic	p-value
treatment	95.21	3	31.74	11.5	0.02
error	11.04	4	2.76		
total	106.2	7			

treatments pair	Tukey HSD Q statistic	Tukey HSD p-value	Tukey HSD inferfence
A vs B	2.788	0.331	insignificant
A vs C	1.522	0.712	insignificant
A vs D	7.825	0.018	* p<0.05
B vs C	1.266	0.796	insignificant
B vs D	5.037	0.076	insignificant
C vs D	6.303	0.037	* p<0.05

P. alvei
50%

source	sum of	degrees of	mean square	F statistic	p-value

	squares SS	freedom vv	MS		
treatment	109.3	3	36.44	6.59	0.05
error	22.12	4	5.53		
total	131.5	7			

P. alvei
25%

source	sum of squares SS	degrees of freedom vv	mean square MS	F statistic	p-value
treatment	57.25	3	19.08	5.643	0.064
error	13.53	4	3.382		
total	70.78	7			

P. alvei
12.5%

source	sum of squares SS	degrees of freedom vv	mean square MS	F statistic	p-value
treatment	224.1	3	74.7	33.54	0.003
error	8.909	4	2.227		
total	233	7			

treatments pair	Tukey HSD Q statistic	Tukey HSD p-value	Tukey HSD inferfence
A vs B	0.526	0.9	insignificant
A vs C	6.818	0.029	* p<0.05
A vs D	11.55	0.004	** p<0.01
B vs C	7.344	0.022	* p<0.05
B vs D	12.08	0.004	** p<0.01
C vs D	4.735	0.091	insignificant

M. plutonius 50%

source	sum of squares SS	degrees of freedom vv	mean square MS	F statistic	p-value
treatment	82.05	3	27.35	6.387	0.053
error	17.13	4	4.282		
total	99.18	7			

M. plutonius 25%

source	sum of squares SS	degrees of freedom vv	mean square MS	F statistic	p-value
treatment	65.52	3	21.84	4.008	0.107
error	21.8	4	5.449		

total	87.32	7	

M. plutonius 12.5%

source	sum of squares SS	degrees of freedom νν	mean square MS	F statistic	p-value
treatment	74.3519	3	24.784	7.1616	0.0437
error	13.8428	4	3.4607		
total	88.1946	7			

treatments pair	Tukey HSD Q statistic	Tukey HSD p-value	Tukey HSD inferfence
A vs B	2.7136	0.3483996	insignificant
A vs C	0.7047	0.8999947	insignificant
A vs D	3.798	0.1667711	insignificant
B vs C	3.4183	0.2153695	insignificant
B vs D	6.5116	0.0333729	* p<0.05
C vs D	3.0933	0.2688377	insignificant
